# Aromatic Character and Relative Stability of Pyrazoloporphyrin Tautomers and Related Protonated Species: Insights into How Pyrazole Changes the Properties of Carbaporphyrinoid Systems

**DOI:** 10.3390/molecules28062854

**Published:** 2023-03-22

**Authors:** Deyaa I. AbuSalim, Timothy D. Lash

**Affiliations:** Department of Chemistry, Illinois State University, Normal, IL 61790-4160, USA

**Keywords:** carbaporphyrins, *N*-confused porphyrins, pyrazole, aromaticity, nucleus independent chemical shifts, anisotropy of induced current density, tautomers, protonation, DFT

## Abstract

Pyrazoloporphyrins (PzPs), which are porphyrin analogues incorporating a pyrazole subunit, are examples of carbaporphyrin-type structures with a carbon atom within the macrocyclic cavity. DFT calculations were used to assess a series of 17 PzP tautomers, nine monoprotonated species and four related diprotonated PzP dications. The geometries of the structures were optimized using M06-2X/6-311++G(d,p), and the relative stabilities computed with the cc-PVTZ functional. Nucleus independent chemical shifts, both NICS(0) and NICS(1)*_zz_*, were calculated, and the anisotropy of the induced current density (AICD) plots were generated for all of the species under investigation. The results for free base PzPs show that fully aromatic PzP tautomers are not significantly more stable than weakly aromatic cross-conjugated species. In addition, strongly aromatic structures with internal CH_2′_s are much less stable, a feature that is also seen for protonated PzPs. The degree of planarity for the individual macrocycles does not significantly correlate with the stability of these structures. The results allow significant aromatic conjugation pathways to be identified in many cases, and provide insights into the aromatic properties of this poorly studied system. These investigations also complement experimental results for PzPs and emphasize the need for further studies in this area.

## 1. Introduction

**Figure 1:**
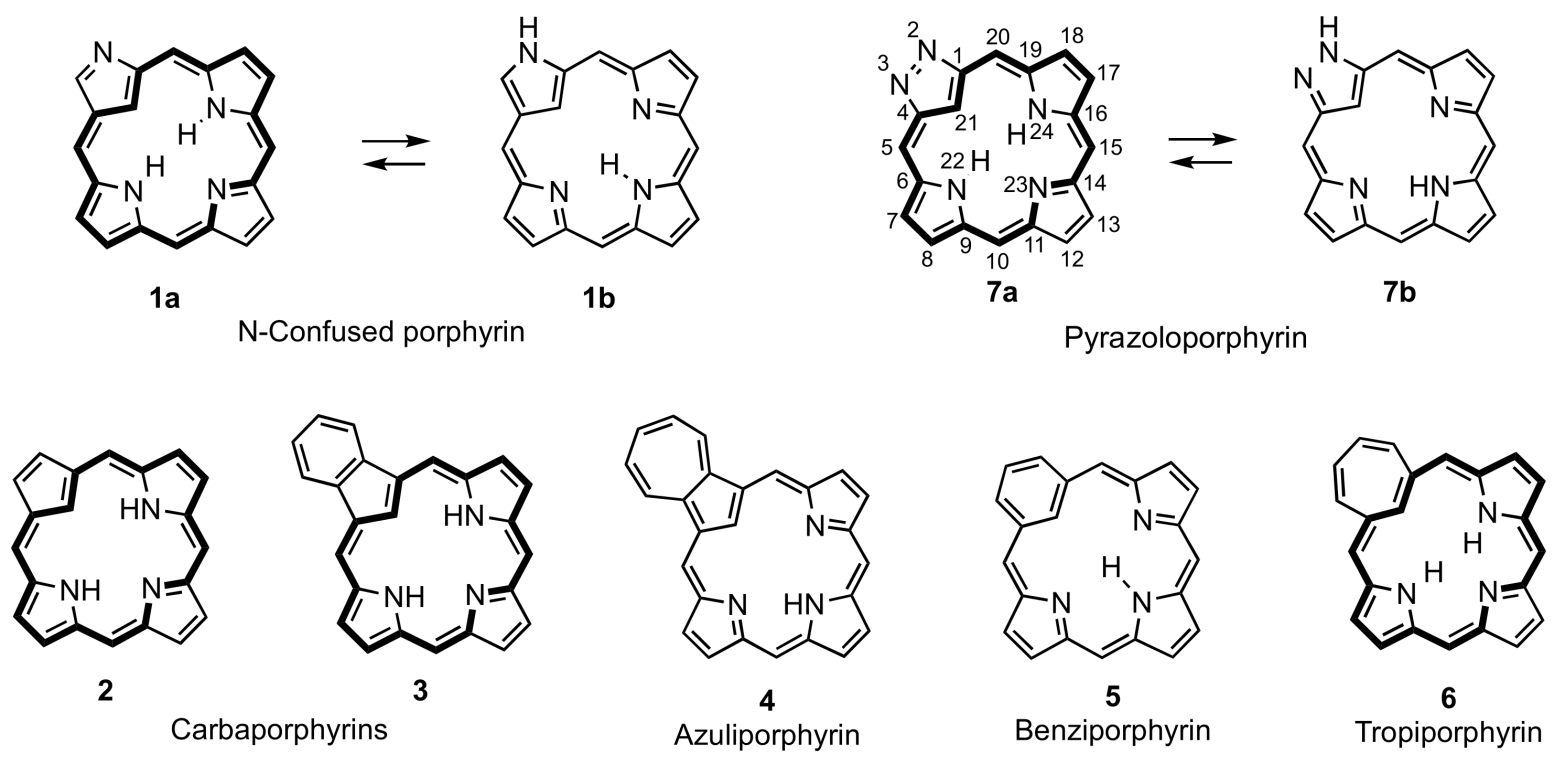
Selected carbaporphyrinoid systems.

Investigations into porphyrin-type structures has exploded over the last thirty years [1,2,3]. In 1994, two groups independently discovered *N*-confused porphyrins (NCPs, **1**) (Figure 1) as by-products in the acid-catalyzed condensation of pyrrole with aromatic aldehydes [4,5], and an efficient synthesis of this system was subsequently reported by Lindsey and Geier [6]. Immediately after the initial disclosure of NCPs, a number of related porphyrinoids were reported, including carbaporphyrins **2** and **3** [7,8,9], azuliporphyrins **4** [10,11], benziporphyrins **5** [12,13,14] and tropiporphyrins **6** [15,16]. Carbaporphyrinoid systems, porphyrin-like molecules with one or more carbon atoms within the macrocyclic core, exhibit diverse and unusual reactivity and may exist as aromatic, nonaromatic or antiaromatic species [1,17,18]. In addition, carbaporphyrin-type systems react with many late transition metal cations to generate stable organometallic derivatives under mild conditions [19,20,21,22]. *N*-Confused porphyrins have been particularly well studied with regard to their metalation properties [22], but complementary organometallic chemistry has also been described for related systems [19,20]. *N*-Confused porphyrins have two major tautomeric forms, **1a** and **1b**, that have dramatically different spectroscopic properties. Tautomer **1a**, which is favored in nonpolar solvents including chloroform, is fully aromatic, and the proton NMR spectra for NCPs exhibit a strong diamagnetic ring current that results in the inner CH being shifted upfield to approximately −4 ppm. Tautomer **1b** is approximately 5 kcal/mol higher in energy [23,24,25,26,27], but is favored in polar aprotic solvents such as DMF or DMSO [23]. This is due to favorable hydrogen bonding interactions between the solvent and the external NH. As **1b** is cross-conjugated, the global aromatic character for the macrocycle is greatly diminished.

**Figure 2:**
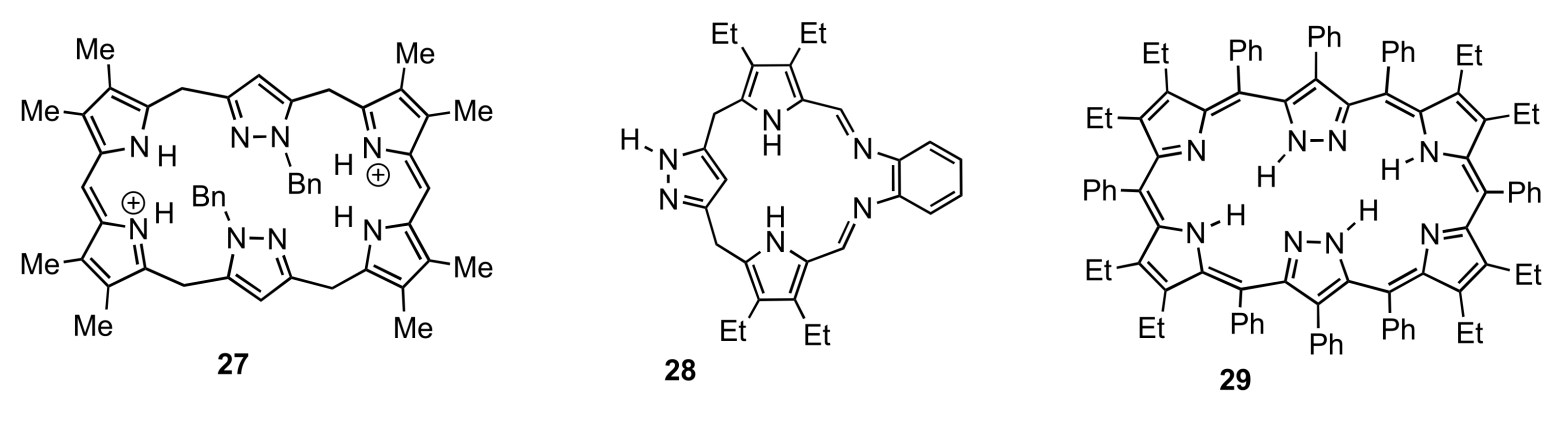
Expanded pyrazole-containing porphyrinoids.

**Scheme 1:**
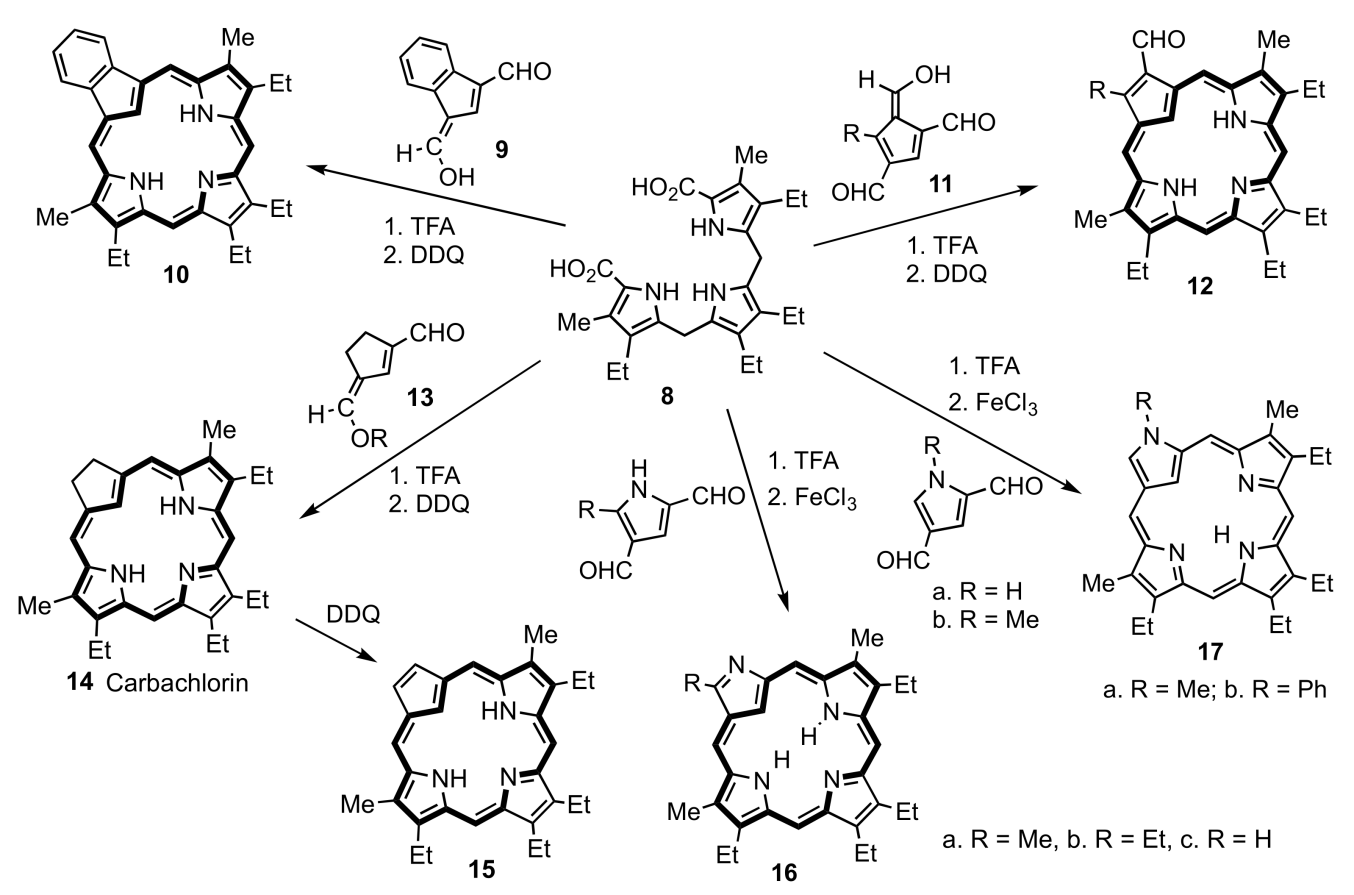
MacDonald ‘3 + 1’ syntheses of carbaporphyrins and *N*-confused porphyrins.

**Scheme 2:**
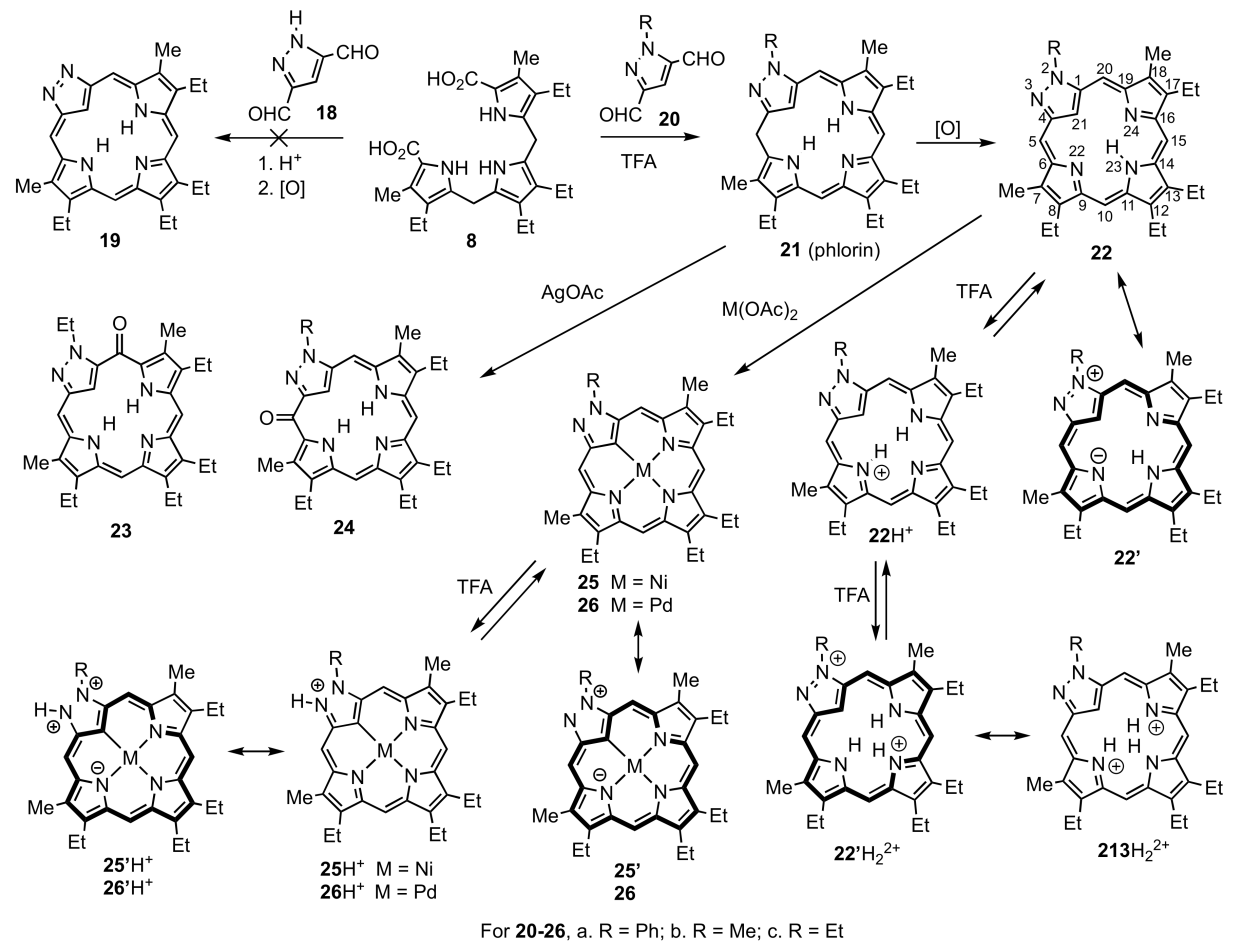
Synthesis and reactivity of *N*-substituted pyrazoloporphyrins.

**Scheme 3:**
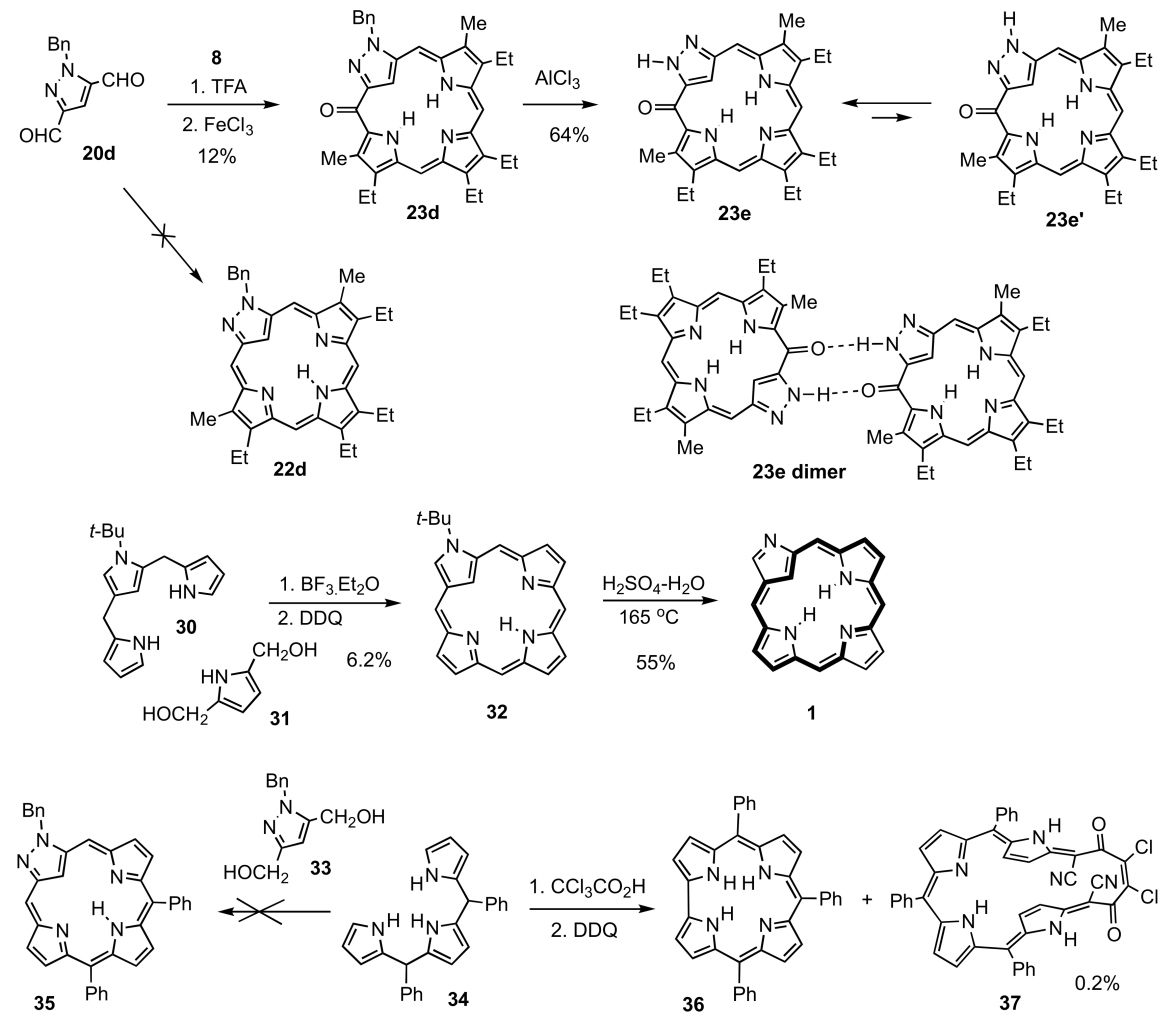
Attempted syntheses of *N*-unsubstituted pyrazoloporphyrins.

Carbaporphyrin **2** is structurally similar to NCP tautomer **1b** and also exhibits a strongly aromatic character [8]. In order to further investigate these systems, porphyrin analogues **7** with pyrazole subunits were targeted for investigation [28,29]. Structures **2**, **1** and **7** provide a series of carbaporphyrinoids with an increasing number of nitrogens in the modified subunit. Therefore, this series can provide new insights into the properties of these systems. In addition, pyrazoloporphyrins (PzPs) might exist in a number of tautomeric forms, including tautomers **7a** and **7b**, that are analogous to the major species for NCP. Although *N*-confused porphyrins are commonly synthesized using Rothemund-Lindsey reaction conditions [4,5,6], alternative routes to NCPs have been developed, including ‘2 + 2’ [30] and ‘3 + 1’ strategies [31,32]. The ‘3 + 1’ version of the MacDonald condensation has been a particularly useful method for preparing carbaporphyrinoid systems [33,34] (Scheme 1). The reaction of tripyrrane **8** with indene dialdehyde **9** in the presence of trifluoroacetic acid (TFA) afforded carbaporphyrin **10**, while trialdehyde **11** gave modest yields of carbaporphyrin aldehydes **12** [7,35]. Methoxymethylene cyclopentene aldehyde **13** similarly afforded carbachlorin **14**, and this can be further oxidized to carbaporphyrin **15** [8]. The same methodology was successfully applied to the preparation of *N*-confused porphyrins **16** and **17** [31,32]. Unfortunately, attempts to condense pyrazole dialdehyde **18** with **8** failed to give the expected PzP **19** (Scheme 2). However, *N*-substituted dialdehydes **20** reacted with **8** to give phlorin **21** and careful oxidation with aqueous FeCl_3_ afforded PzPs **22** [28,29] The presence of *N*-phenyl, methyl or ethyl substituents prevents the system from tautomerizing into a fully aromatic porphyrinoid. The proton NMR spectra for PzPs **22** showed that they possess a weak diatropic ring current, and this was attributed to contributions from dipolar resonance contributors such as **22′**. The sequential protonation of **22** with TFA initially gave monocation **22**H^+^ and then dication **22**H_2_^2+^. Oxidation of the phlorin intermediate with silver(I) acetate gave oxopyrazolophlorins **23** and **24**. Metalation with nickel(II) or palladium(II) acetate afforded the corresponding organometallic derivatives **25** and **26**, respectively [28,29]. The proton NMR spectra for **25** and **26** showed the presence of moderate diatropic ring currents. However, all vestiges of an aromatic character were lost upon protonation. The aromatic character of **25** and **26** can be attributed to dipolar resonances **25**H^+^ and **26**H^+^ that incorporate 18π electron delocalization pathways. However, protonation of the external nitrogens inhibits this interaction because the corresponding canonical forms **25′**H^+^ and **26′**H^+^ would necessitate the placement of positive charges on adjacent nitrogen atoms. The earliest investigations into pyrazole-containing porphyrinoids were directed at the synthesis of non-aromatic hexaphyrin analogues **27** [36], and texaphyrin analogue **28** has also been reported [37]. More significantly, a fully conjugated hexaphyrin analogue designated Siamese-twin porphyrin **29** has been synthesized [38] (Figure 2). This system proved to be nonaromatic but possesses two binding pockets that are similar to the binding cores of porphyrins. Extensive metalation studies on this system have been reported [39]. Furuta also investigated the synthesis of PzPs [40] (Scheme 3). Tripyrrane **8** was condensed with pyrazole dialdehyde **20d** in the presence of trifluoroacetic acid, followed by oxidation with FeCl_3_, but in this case oxopyrazolophlorin **23d** was isolated instead of the expected *N*-benzyl PzP **22d**. Cleavage of the benzyl group with AlCl_3_ afforded a tautomeric mixture of *N*-unsubstituted oxopyrazolophlorins **23e** and **23e′** [40]. The favored tautomer **23e** self-assembled as a hydrogen bonded dimer (Scheme 3). Tripyrrane analogues can be reacted with dialcohols to give modified porphyrinoids. For instance, *tert*-butyl *N*-confused tripyrrane **30** reacted with pyrrole dicarbinol **31** to give *tert*-butyl NCP **32** and subsequent treatment with aqueous sulfuric acid at 160 °C afforded unsubstituted *N*-confused porphyrin **1** [41]. However, the acid-catalyzed condensation of pyrazole dialcohol **33** with diphenyltripyrrane **34**, followed by oxidation with 2,3-dichloro-5,6-dicyano-1,4-benzoquinone (DDQ), did not give targeted PzP **35**, but instead afforded a mixture of triphenylcorrole **36** and a nonaromatic expanded porphyrinoid **37** that incorporated a 6-carbon chain derived from DDQ [40].

Due to the many difficulties encountered in the synthesis of pyrazoloporphyrins, this system is not well studied. *N*-Unsubstituted PzPs are presently unknown, and it has not been possible to contrast their tautomeric properties with closely related NCPs. In order to gain a better understanding of the pyrazoloporphyrin system, a computational study was conducted. The goals were threefold. Firstly, the conformations and relative stabilities of 17 potential PzP tautomers were considered. Secondly, the aromatic characteristics of these tautomers were assessed using nucleus independent chemical shift (NICS) calculations and the anisotropy of induced current density (AICD) plots. Thirdly, these methods were used to predict the relative stabilities and aromatic characteristics of protonated PzPs.

## 2. Results

### 2.1. Aromatic and Nonaromatic Tautomers of Pyrazoloporphyrins

Computational methods can provide valuable insights into the structures and aromatic properties of porphyrinoid systems [42,43,44,45,46,47,48,49,50]. In our earlier work, we have assessed the relative stabilities and aromatic properties of a wide range of tautomeric structures and protonated species for carbaporphyrins [51], benzo- and naphthocarbaporphyrins [52,53], heterocarbaporphyrins [54], carbachlorins [8,55], dicarbaporphyrins [56,57,58], benziporphyrins [59], pyreniporphyrins [60], neo-confused porphyrins [52,61], azuliporphyrins [62], 21-oxyazuliporphyrins [63], tropiporphyrins [62], oxyquinoliziniporphyrins [64] and carbatriphyrins [65]. In this study, a series of 17 free base PzP tautomers (Table 1 and Table 2) were examined. The structures were optimized using M06-2X with the triple-ζ basis set 6-311++G(d,p). Seven tautomers are fully conjugated (Table 1), or contain a cross-conjugated element, while the 10 remaining tautomers have methylene bridges (Table 2) that interrupt macrocyclic conjugation. The relative stability and π-delocalization pathways in these tautomers were investigated. The peripheral atoms for PzP are numbered 1–20, and the inner atoms are designated 21–24, as shown for structure **7a** in Figure 1. Two hydrogens are relocated in each of the tautomeric structures, and the numerical positions of these hydrogens are designated in the structure notation. For instance, the most stable tautomer of pyrazoloporphyrin is **PzP-22,24-H**, where the “mobile” hydrogens are located at positions 22 and 24.

Tautomer **PzP-22,24-H** is very close in energy to the cross-conjugated form **PzP-2,23-H** (Table 1). This differs from NCPs, where the equivalent aromatic tautomer is approximately 5 kcal/mol lower in energy than the cross-conjugated version. It is noteworthy that the relative energies and Gibbs free energies are very similar for all of the calculated tautomers, demonstrating that entropic factors are not significant. As is the case for aromatic carbaporphyrins and NCPs, PzP has three internal hydrogens, and the crowded cavity leads to the pyrazole ring being pivoted out of the macrocyclic plane (Table 3). Nevertheless, some distortion to the macrocycle is present in **PzP-2,23-H** as well. Related tautomers **PzP-22,23-H**, **PzP-2,22-H** and **PzP-2,24-H** are 6.67–7.88 kcal/mol higher in energy than **PzP-22,24-H** (Table 1). This is due to less effective hydrogen bonding interactions within the core [66,67,68,69], as well as less than optimal placement of the hydrogens leading to unfavorable steric interactions. Two additional fully conjugated forms with internal methylene units, **PzP-21,22-H** and **PzP-21,23-H**, are possible, but these are 18.32 and 11.27 kcal/mole higher in energy, respectively, than **PzP-22,24-H**. The former is less stable due to the core arrangement being less well suited for hydrogen bonding interactions as well as placing the internal hydrogens at adjacent positions. Nevertheless, both tautomers are virtually planar and possess 18π electron conjugation pathways [70]. This result demonstrates that global aromaticity and overall planarity are not in themselves sufficient to determine the stability of these structures. It is noteworthy that only one example of a carbaporphyrinoid system with an internal CH_2_ is presently known [57]. Tautomers with interrupted conjugation due to the presence of a methylene bridge (Table 2) were all much less stable with relative energies of 22.28–32.76 kcal/mol higher than **PzP-22,24-H**. The degree of distortion for these structures varies considerably, although **PzP-2,5-H**, **PzP-2,10-H**, **PzP-2,15-H**, **PzP-2,20-H** and **PzP-5,23-H** are all essentially planar. This factor does not play a role in determining stability, although the first four are particularly unstable.

**Figure 3:**
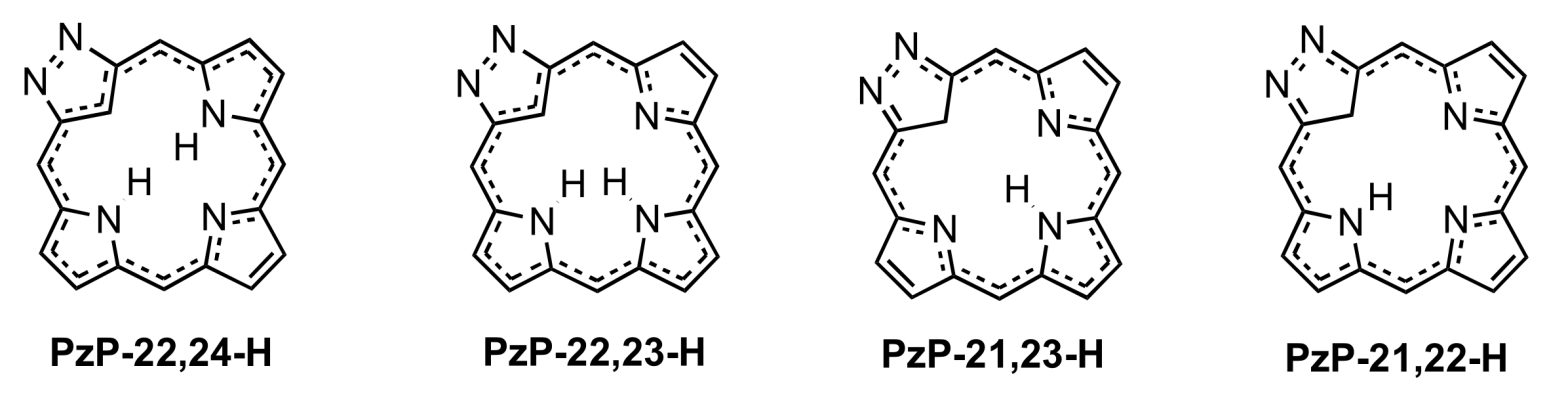
Proposed aromatic circuits in selected pyrazoloporphyrin tautomers.

The aromatic character of the individual tautomers were probed using nucleus independent chemical shift (NICS) calculations and the anisotropy of induced current density (AICD) plots. NICS calculations [71] were carried out using the GIAO method. NICS calculations assess the chemical shifts that might be observed in proton NMR studies but does not require a nucleus to be present at the site of interest. Large negative values correspond to highly shielded regions that result from aromatic ring currents, while positive values correspond to deshielded regions. The latter could result from paratropic ring currents (antiaromaticity), but will also emerge when a ring is placed just outside of a major aromatic conjugation pathway. Two types of NICS calculations, NICS(0) and NICS(1)_zz_, were conducted. Standard NICS calculations take into account the effects due to σ and π electrons and do not always reliably assess aromatic properties, but NICS_zz_ primarily measures the effects due to the π system [72,73,74,75]. NICS_zz_ calculations were performed 1 Å above the ring. The two techniques were mostly consistent, but the numerical values obtained for NICS(1)_zz_ are much larger than those obtained using NICS(0). In the discussion, the NICS(1)_zz_ values are emphasized. Favored tautomer **PzP-22,24-H** gave strongly aromatic NICS(0) and NICS(1)_zz_ values of −13.05 and −30.62 ppm, respectively, while cross-conjugated form **PzP-2,23-H** gave very weakly diatropic values of −1.76 and −2.88 ppm. Given that these structures have very similar stabilities, the aromatic nature of **PzP-22,24-H** appears to offer few benefits. **PzP-22,23-H** has comparable aromatic properties to **PzP-22,24-H**, while **PzP-2,22-H** and **PzP-2,24-H** are similar, albeit slightly more diatropic, to **PzP-2,23-H**. High energy tautomers **PzP-21,23-H** and **PzP-21,22-H** with internal CH_2_ units both gave strongly aromatic NICS(1)_zz_ values (Table 1). The NICS(0)/NICS(1)_zz_ values for the individual pyrazole and pyrrole rings in these structures were also determined. These rings were designated a, b, c and d, where ring a corresponds to the pyrazole unit (Table 1 and Table 2). For **PzP-22,24-H**, rings b and d gave NICS(1)_zz_ strongly negative values (ca. −30 ppm) but ring c gave a smaller negative value of −11.73 ppm and ring a was only −2.12 ppm. These results suggest that an 18π electron circuit that passes through the inside of rings a and c and the outside of rings b and d is favored (Figure 3). This type of conjugation pathway is also favored for NCP **1a** and carbaporphyrins **2** and **3** (Figure 1). For tautomer **PzP-2,23-H**, ring a gives a strongly negative value of ca. −20 ppm, indicating that the pyrazole unit is strongly aromatic but primarily isolated from the rest of the macrocycle. This is also seen for tautomers **PzP-2,22-H** and **PzP-2,24-H**. Tautomer **PzP-22,23-H** gives strongly negative values for rings c and d that are consistent with the presence of an 18π electron pathway (Figure 3). Tautomer **PzP-21,23-H** has strongly negative NICS(1)_zz_ values for ring a and c, while **PzP-21,22-H** gives similarly negative values for rings a and d. These results show that 18π electron circuits that pass around the outside of ring a are present (see Figure 3). Tautomers with interrupted conjugation (Table 2) give varying values for the individual rings. In **PzP-2,5-H**, **PzP-2,10-H**, **PzP-2,15-H** and **PzP-2,20-H**, the pyrazole unit gives strongly negative NICS_zz_(1a) values, but the aromatic properties of this ring are diluted in the remaining tautomers.

**Figure 4:**
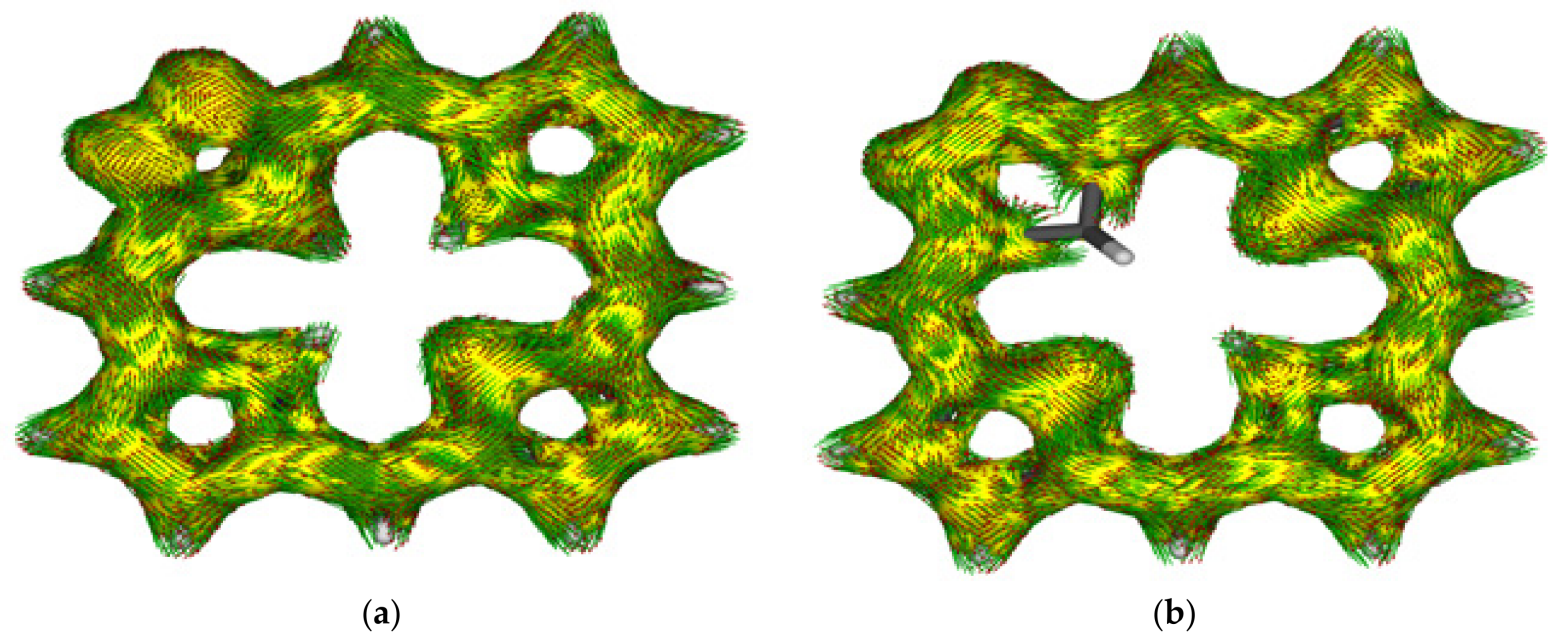
AICD plots (isovalues 0.05) for **PzP-22,24-H** (**a**) and **PzP-21,23-H** (**b**).

**Figure 5:**
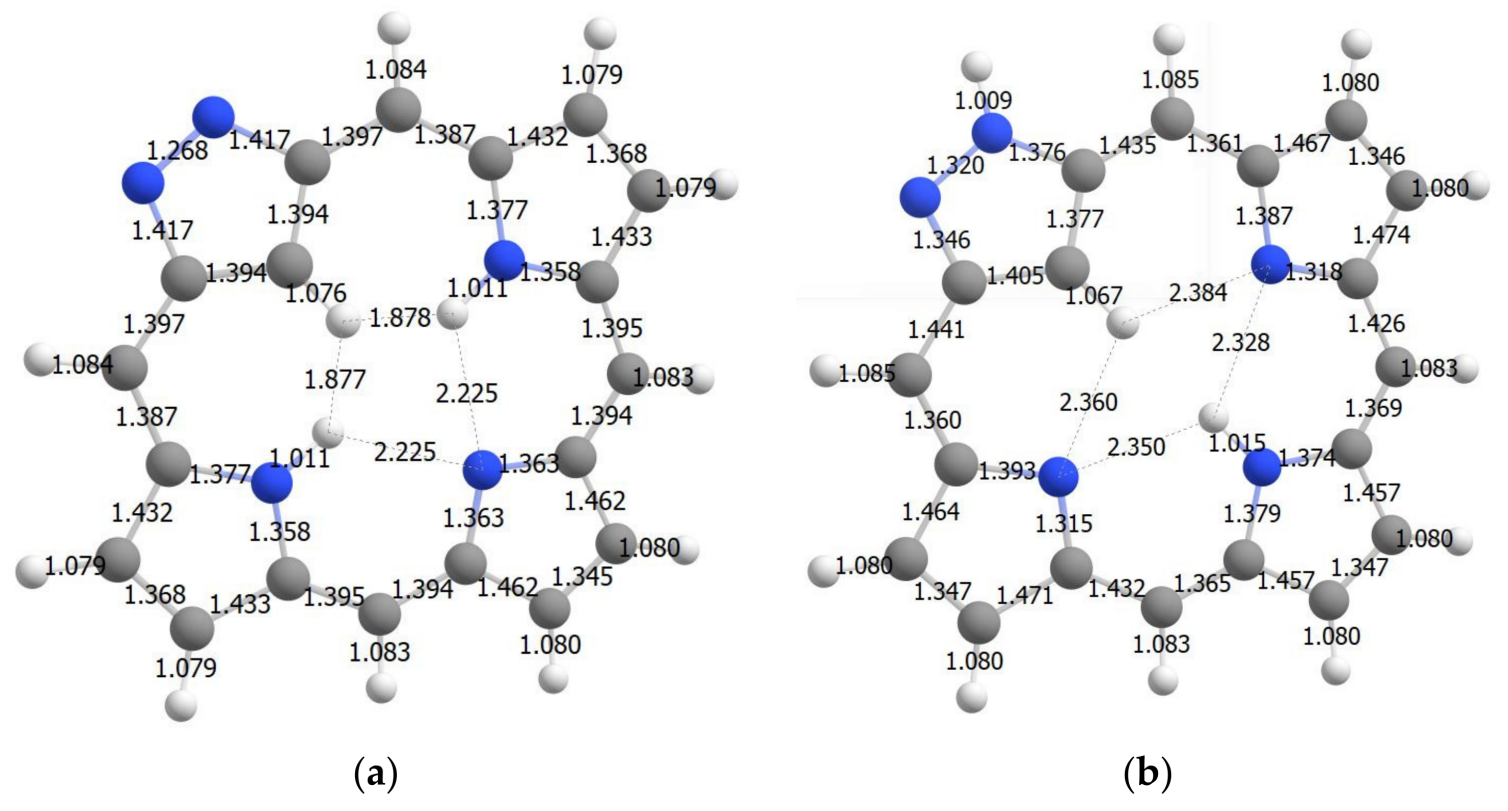
Calculated bond lengths and interatomic distances (Å) for the core atoms in pyrazoloporphyrin tautomers **PzP-22,24-H** (**a**) and **PzP-2,23-H** (**b**).

In addition to the NICS calculations, all the investigated structures were probed using anisotropy of induced current density (AICD) [76] (see Supporting Information). AICD plots show the induced flow of electrons due to an applied magnetic field and allow the visualization of aromatic ring currents. The plot for **PzP-22,24-H** confirms the presence of the proposed 18π electron circuit (Figure 4), but a degree of bifurcation is also present. The main aromatic pathways pass through the inside of the pyrazole ring, but the routes are less well delineated in ring c. This observation is also consistent with the NICS calculations. Unfavored aromatic tautomer **PzP-21,23-H** also shows the presence of an 18π electron pathway, but alternative routes through the pyrrolic subunits are clearly evident. Bond length calculations provide further insights (Figure 5). For **PzP-22,24-H**, the closed circuit representing the proposed aromatic pathway shows little bond length alternation apart from the carbon-carbon bonds within the individual pyrrole units. C6-C7, C7-C8, C16-C17 and C18-C19, all of which fall within the aromatic pathway, gave values of 1.432–1.433 Å, but the equivalent bonds in ring c, C11-C12 and C13-C14, gave slightly longer values of 1.462 Å. C12-C13 for ring c gave a relatively short bond length of 1.345 Å compared to the equivalent bonds C7-C8 and C17-C18 in rings b and d, which came out as 1.368 Å. These trends are consistent with the proposed conjugation pathway. For **PzP-2,23-H**, a substantial bond length alternation is evident, as would be expected for a nonaromatic porphyrinoid.

### 2.2. Mono- and Diprotonated Pyrazoloporphyrins

**Figure 6:**
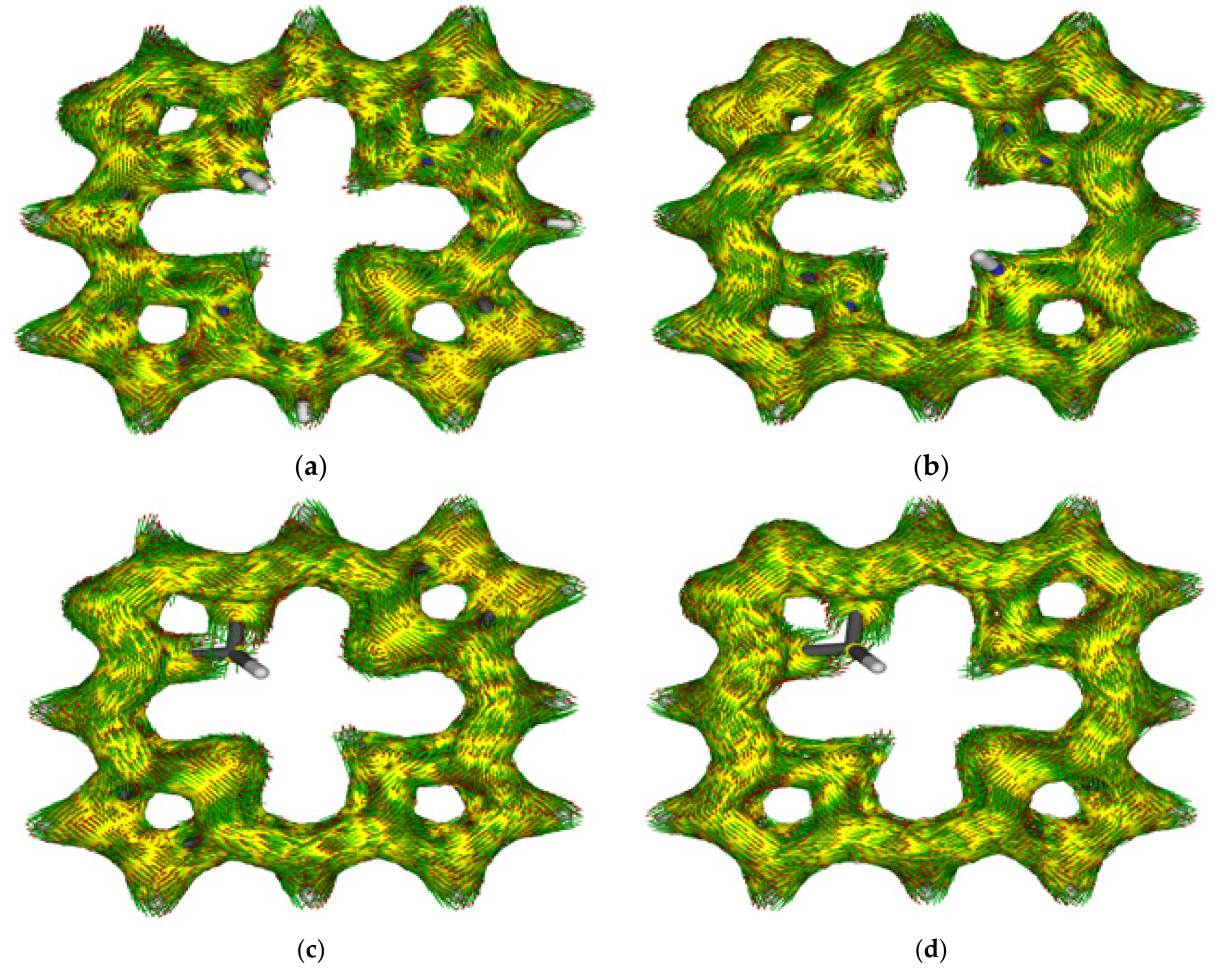
AICD plots (isovalues 0.05) for **PzP-2,22,24-H^+^** (**a**), **PzP-22,23,24-H^+^** (**b**), **PzP-2,21,23-H^+^** (**c**), and **PzP-21,22,24-H^+^** (**d**).

**Figure 7:**
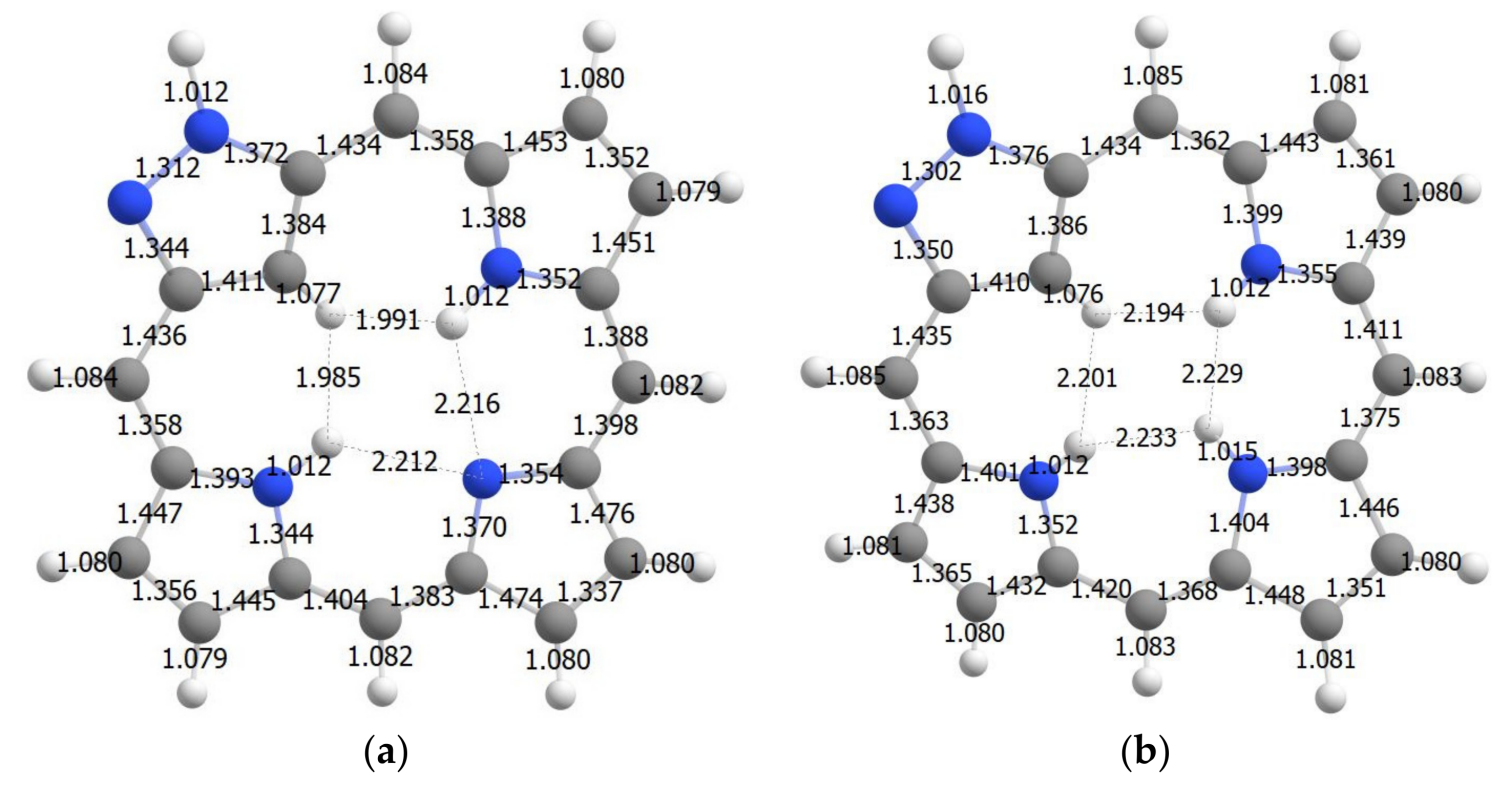
Calculated bond lengths and interatomic distances for the core atoms for pyrazoloporphyrin monocation **PzP-2,22,24-H^+^** (**a**) and dication **[PzP-2,22,23,24-H]^2+^** (**b**).

**Figure 8:**
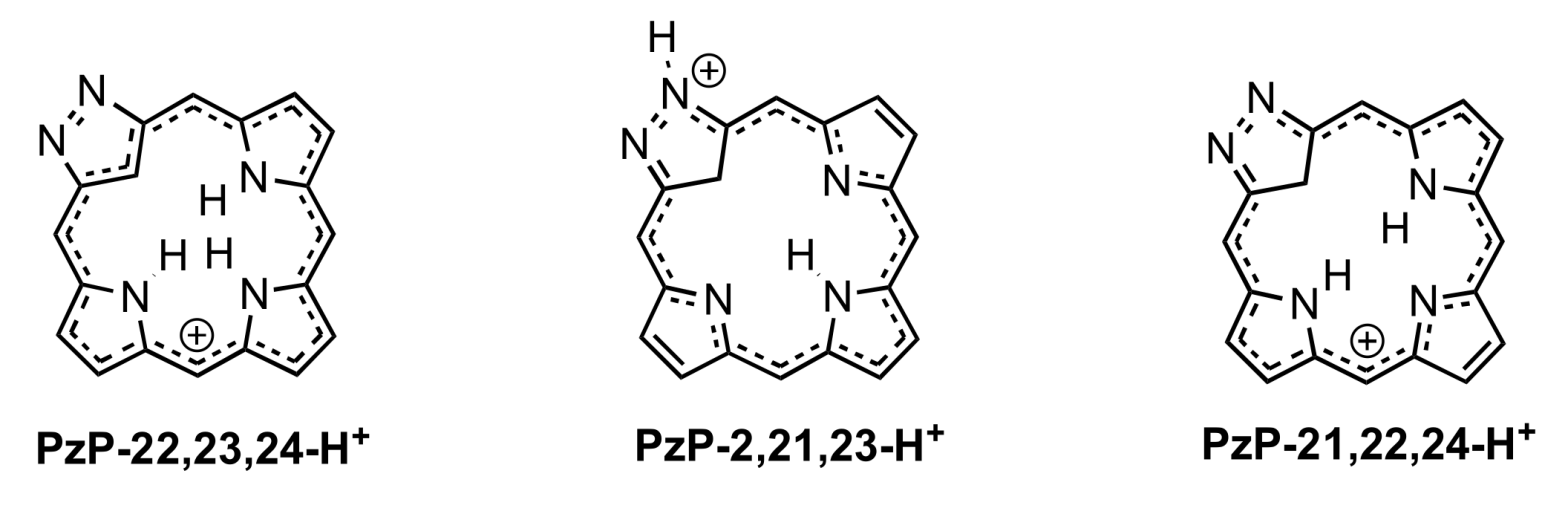
Proposed aromatic circuits in selected pyrazoloporphyrin cations.

A series of 9 monoprotonated pyrazoloporphyrin tautomers were considered (Table 4). For this series, only structures that were fully conjugated were investigated. The most stable tautomer, **PzP-2,22,24-H^+^**, was protonated onto an external nitrogen. The lowest energy tautomer that had been internally protonated, **PzP-22,23,24-H^+^**, was calculated to be nearly 17 kcal/mol higher in energy. The two remaining externally protonated tautomers, **PzP-2,22,23-H^+^** and **PzP-2,23,24-H^+^**, are only approximately 4 kcal/mol higher in energy, and the differences can be attributed to a combination of steric and poorer hydrogen bonding interactions. Five of the monocations have internal methylene units. The most stable of these, **PzP-2,21,23-H^+^**, is still over 17 kcal/mol higher in energy than **PzP-2,22,24-H^+^**. This species is externally protonated, but the most stable version with an inner CH_2_ that is internally protonated, **PzP-21,22,24-H^+^**, is nearly 21 kcal/mol higher in energy. The three most favored tautomers, **PzP-2,22,24-H^+^**, **PzP-2,22,23-H^+^** and **PzP-2,23,24-H^+^**, are by far the least aromatic forms, with NICS(1)*_zz_* values between −4.5 and −8.5 ppm. The AICD plot for **PzP-2,22,24-H^+^** (Figure 6a) does not show clear global aromatic pathways. Furthermore, the calculated bond lengths for **PzP-2,22,24-H^+^** show a considerable amount of bond length alternation (Figure 7). In contrast, the AICD plot for **PzP-22,23,24-H^+^** (Figure 6b) indicates that a 19-atom pathway is favored (Figure 8), and this corresponds to the presence of an 18π electron cationic [19]annulene component. This analysis is supported by the NICS(1)*_zz_* data, as these show large negative values for rings *b*, *c* and *d*, but not for the pyrazole ring. **PzP-2,21,22-H^+^**, which is externally protonated but with an internal methylene unit, gave an AICD plot (Figure 6c) that was consistent with the presence of an 18-atom 18π electron pathway, while the AICD plot for **PzP-21,22,24-H^+^** (Figure 6d), which also has an internal methylene unit but is protonated internally, favors a 19-atom 18π electron circuit (Figure 8). Again, these conjectures are supported by the NICS(1)*_zz_* calculations (Table 5).

Four diprotonated PzP dications were considered. By far the most stable tautomer for this series was **[PzP-2,22,23,24-H]^2+^**, but this species is the least aromatic with a NICS(1)*_zz_* value of only −7.49 ppm. The remaining dications, **[PzP-2,21,22,23,24-H]^2+^**, **[PzP-2,22,23,24-H]^2+^** and **[PzP-2,22,23,24-H]^2+^**, have internal CH_2_ units and NICS(1)*_zz_* values between −24.93 and −29.07 ppm. The calculated bond lengths for **[PzP-2,22,23,24-H]^2+^** show extensive bond length alternation (Figure 7), as would be expected for a weakly aromatic species.

## 3. Computational Methods

All DFT calculations used the Gaussian 16 revision C.01 [77]. Geometry optimizations were performed using M06-2X/6-311++G(d,p) [78,79,80]. Using the same functional and basis set, the corresponding vibrational frequencies were computed to confirm the absence of imaginary frequencies and derive zero-point energy and vibrational entropy corrections from unscaled frequencies. Single point energy calculations were performed on the optimized minima using M06-2X/cc-PVTZ [81]. NICS values were calculated using the GIAO method, and AICD plots were obtained from CGST calculations. NICS(0) was calculated at the mean position of the four heavy atoms at the center of the macrocycle. NICS(*a*), NICS(*b*), NICS(*c*), and NICS(*d*) were calculated at the mean position of the five atoms that make up each component ring in the macrocycle. Additionally, NICS(1)*_zz_*, NICS(*1a*)*_zz_*, NICS(*1b*)*_zz_*, NICS(*1c*)*_zz_*, and NICS(*1d*)*_zz_* were obtained by applying the same calculations 1 Å above each of the corresponding points extracting the zz contribution of the magnetic tensor. The resulting energies, cartesian coordinates, and AICD plots can be found in the Supplementary Materials.

## 4. Conclusions

Pyrazoloporphyrins (PzPs) represent a logical extension of the structural concepts associated with *N*-confusion, but to date research on this system has been limited, and *N*-unsubstituted pyrazoloporphyrins are not presently known. DFT calculations have been used to assess the properties of *N*-unsubstituted PzPs, and the results provide new and unexpected insights. The aromatic properties were probed using anisotropy of induced current density (AICD) plots and nucleus independent chemical shift (NICS) calculations. The two most stable tautomers of PzP differ in energy by <0.4 kcal/mol, even though one form is strongly aromatic, while the other version is cross-conjugated and only weakly aromatic. Tautomers with internal CH_2_ units are also very aromatic species, but have much higher calculated energies. Monoprotonation can give rise to a number of monocationic species, nine of which were considered. The favored tautomers are externally protonated with very weak diatropic characteristics. Internal protonation gives a cation that is strongly aromatic, but it is nearly 17 kcal/mol higher in energy than the most favorable externally protonated form. Cations with internal methylenes are also very aromatic species but are relatively unstable. Diprotonated PzP dications were also considered. The most stable dication is weakly aromatic, and fully aromatic cations with inner CH_2′_s are >15 kcal/mol higher in energy. The results are in accord with the reported properties for 2-substituted PzPs, and highlight the profoundly altered properties of this system compared to NCPs and carbaporphyrins.

## Figures and Tables

**Table 1:** Calculated Relative Energies (kcal/mol) and NICS Values (ppm) for Continuously Conjugated Free Base Unsubstituted Pyrazoloporphyrin Tautomers.

	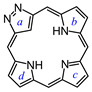 PzP-22,24-H	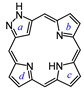 PzP-2,23-H	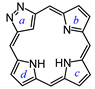 PzP-22,23-H	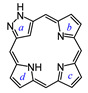 PzP-2,22-H	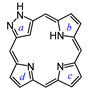 PzP-2,24-H	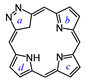 PzP-21,22-H	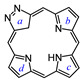 PzP-21,23-H
ΔG298 (kcal/mol) ΔE (kcal/mol)	0.00	−0.25	6.20	7.24	6.42	16.21	9.17
	0.00	0.37	6.67	7.88	7.08	18.32	11.27
NICS(0) **NICS(01)_zz_**	−13.05	−1.76	−11.88	−2.49	−2.20	−11.96	−11.08
	**−30.62**	**−2.88**	**−28.46**	**−5.85**	**−4.87**	**−29.02**	**−27.24**
NICS(a) **NICS(a1)_zz_**	+1.26	−8.96	+03.59	−9.87	−9.69	−15.12	−13.57
	**+2.12**	**−19.84**	**+04.64**	**−23.46**	**−22.70**	**−49.64**	**−46.92**
NICS(b) **NICS(b1)_zz_**	−11.73	−0.04	−2.83	+0.65	−4.05	+1.24	−0.76
	**−31.37**	**−5.45**	**−13.35**	**−6.40**	**−10.74**	**−5.94**	**−10.11**
NICS(c) **NICS(c1)_zz_**	−2.03	−3.97	−10.56	+1.02	+0.40	−2.24	−12.94
	**−11.73**	**−10.70**	**−25.72**	**−5.05**	**−6.39**	**−13.27**	**−33.60**
NICS(d) **NICS(d1)_zz_**	−11.72	−0.82	−13.19	−4.33	−0.43	−12.70	−0.76
	**−31.34**	**−7.07**	**−37.48**	**−11.71**	**−8.75**	**−33.32**	**−10.11**

**Table 2:** Calculated Relative Energies (kcal/mol) and NICS Values (ppm) for Pyrazoloporphyrin Tautomers with Methylene Bridges.

	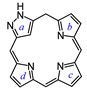 PzP-2,20-H	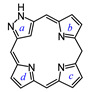 PzP-2,15-H	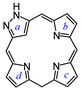 PzP-2,10-H	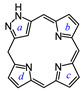 PzP-2,5-H	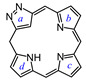 PzP-5,22-H
ΔG298 (kcal/mol) ΔE (kcal/mol)	28.14	27.38	27.56	28.43	27.27
	32.15	31.49	31.67	32.76	30.13
NICS(0) **NICS(01)_zz_**	+0.72	+1.09	+1.09	+0.53	−0.55
	**+5.25**	**+5.76**	**+5.72**	**+4.52**	**+1.87**
NICS(a) **NICS(a1)_zz_**	−10.05	−9.11	−9.01	−9.72	+1.94
	**−24.53**	**−22.74**	**−22.69**	**−24.66**	**−7.33**
NICS(b) **NICS(b1)_zz_**	+1.50	+0.34	−0.48	−1.01	−1.04
	**−4.15**	**−5.95**	**−7.19**	**−8.46**	**−9.17**
NICS(c) **NICS(c1)_zz_**	−0.06	+0.97	+1.41	+0.50	−3.85
	**−7.02**	**−5.31**	**−4.36**	**−5.85**	**−15.13**
NICS(d) **NICS(d1)_zz_**	−1.73	−1.18	−0.36	+2.40	−9.23
	**−10.05**	**−8.76**	**−7.55**	**−2.36**	**−23.78**
	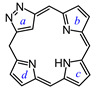 **PzP-5,23-H**	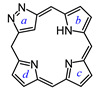 **PzP-5,24-H**	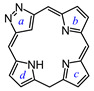 **PzP-10,22-H**	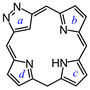 **PzP-10,23-H**	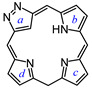 **PzP-10,24-H**
ΔG298 (kcal/mol) ΔE (kcal/mol)	19.93	23.23	24.78	21.06	22.96
	22.28	25.75	28.03	24.21	25.85
NICS(0) **NICS(01)_zz_**	−0.42	+0.16	−0.08	+0.36	−0.09
	**+0.64**	**+2.89**	**+0.74**	**+1.84**	**+1.92**
NICS(a) **NICS(a1)_zz_**	+3.14	+1.24	−0.06	+2.34	+1.14
	**−4.64**	**−8.70**	**−11.70**	**−7.05**	**−8.67**
NICS(b) **NICS(b1)_zz_**	−2.24	−9.41	−1.89	−3.85	−9.65
	**−11.55**	**−22.39**	**−11.35**	**−15.25**	**−22.94**
NICS(c) **NICS(c1)_zz_**	−9.64	−1.76	−1.15	−9.31	−0.72
	**−23.17**	**−10.12**	**−9.94**	**−21.91**	**−8.50**
NICS(d) **NICS(d1)_zz_**	−0.47	+01.05	−9.71	−1.63	−0.55
	**−8.40**	**−5.19**	**−23.77**	**−11.44**	**−7.54**

**Table 3:** Calculated dihedral angles for the individual pyrrole and pyrazole rings within a series of pyrazoloporphyrin tautomers and related protonated species.

Molecule	*ab*	*bc*	*cd*	*da*	Average
**PzP-22,24-H**	13.43	−1.41	1.39	−13.41	7.41
**PzP-22,23-H**	−10.36	−0.04	−6.48	16.13	8.25
**PzP-2,23-H**	9.94	0.95	−1.54	−9.73	5.54
**PzP-2,22-H**	−7.75	−1.53	−0.67	10.15	5.03
**PzP-2,24-H**	10.93	−0.08	−2.37	−8.09	5.37
**PzP-2,20-H**	0.00	0.00	0.00	0.00	0.00
**PzP-2,15-H**	0.01	0.00	0.00	0.00	0.00
**PzP-2,10-H**	0.00	0.00	0.01	0.00	0.00
**PzP-2,5-H**	−0.01	0.01	−0.01	0.13	0.04
**PzP-5,22-H**	−12.70	1.67	−4.71	20.63	9.93
**PzP-5,23-H**	0.03	0.00	−0.01	−0.03	0.02
**PzP-5,24-H**	11.87	−1.58	−2.74	−10.12	6.58
**PzP-10,22-H**	−7.46	0.16	−2.48	8.85	4.74
**PzP-10,23-H**	−10.59	−3.18	3.77	12.83	7.59
**PzP-10,24-H**	−9.69	1.95	0.87	8.90	5.35
**PzP-21,22-H**	0.00	0.00	0.00	0.00	0.00
**PzP-21,23-H**	0.00	0.01	−0.01	−0.01	0.01
**[PzP-22,23,24-H]^+^**	19.03	−13.81	13.83	−19.05	16.43
**[PzP-2,22,24-H]^+^**	−18.70	2.12	−1.69	18.15	10.17
**[PzP-2,22,23-H]^+^**	15.98	−0.81	7.48	−20.99	11.31
**[PzP-2,22,24-H]^+^**	21.07	−7.64	0.32	−15.37	11.10
**[PzP-21,22,24-H]^+^**	0.02	−0.02	0.19	−0.21	0.11
**[PzP-21,22,23-H]^+^**	−1.79	−2.69	10.82	−8.06	5.84
**[PzP-2,21,22-H]^+^**	0.00	0.00	0.00	0.00	0.00
**[PzP-2,21,23-H]^+^**	0.01	−0.02	0.03	−0.02	0.02
**[PzP-2,21,24-H]^+^**	0.00	0.00	0.00	0.00	0.00
**[PzP-2,22,23,24-H]^2+^**	−23.25	15.19	−14.96	22.85	19.06
**[PzP-2,21,22,24-H]^2+^**	−0.01	0.01	−0.01	0.01	0.01
**[PzP-2,21,23,24-H]^2+^**	−7.10	12.00	−4.20	−0.93	6.06
**[PzP-2,21,22,23-H]^2+^**	1.56	3.13	−11.82	6.83	5.84

**Table 4:** Calculated Relative Energies (kcal/mol) and NICS Values (ppm) for Monoprotonated Pyrazoloporphyrin Cations.

	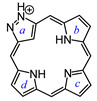 [PzP-2,22,24-H]^+^	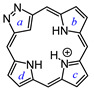 [PzP-22,23,24-H]^+^	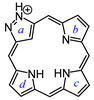 [PzP-2,22,23-H]^+^	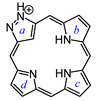 [PzP-2,23,24-H]^+^	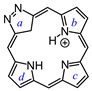 [PzP-21,22,24-H]^+^
ΔG298 (kcal/mol) ΔE (kcal/mol)	0.00	16.70	3.46	4.07	−19.01
	0.00	16.97	3.65	4.29	20.89
NICS(0) **NICS(01)_zz_**	−4.50	−12.54	−2.72	−2.99	−15.01
	**−8.55**	**−29.17**	**−4.52**	**−6.91**	**−36.42**
NICS(a) **NICS(a1)_zz_**	−11.38	+3.39	−9.79	−9.40	−19.92
	**−19.15**	**+6.46**	**−18.49**	**−20.79**	**−60.83**
NICS(b) **NICS(b1)_zz_**	−5.09	−12.81	−0.22	−5.64	−11.30
	**−14.15**	**−39.27**	**−5.89**	**−13.45**	**−31.17**
NICS(c) **NICS(c1)_zz_**	−2.43	−12.02	−4.43	−4.99	−0.13
	**−0.95**	**−23.71**	**−11.56**	**−14.65**	**−13.29**
NICS(d) **NICS(d1)_zz_**	−5.97	−12.80	−6.15	−1.30	−11.30
	**−16.03**	**−39.26**	**−17.63**	**−10.77**	**−31.16**
	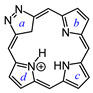 **[PzP-21,22,23-H]^+^**	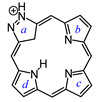 **[PzP-2,21,22-H]^+^**	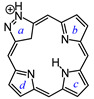 **[PzP-2,21,23-H]^+^**	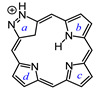 **[PzP-2,21,24-H]^+^**	
ΔG298 (kcal/mol) ΔE(kcal/mol)	23.94	23.96	14.90	22.71	
	25.84	26.52	17.29	24.87	
NICS(0) **NICS(01)_zz_**	−12.55	−12.40	−10.92	−8.67	
	**−29.99**	**−30.19**	**−26.83**	**−19.98**	
NICS(a) **NICS(a1)_zz_**	−16.02	−21.54	−19.10	−15.79	
	**−51.77**	**−52.96**	**−47.98**	**−39.29**	
NICS(b) **NICS(b1)_zz_**	+1.65	+6.16	+4.60	−6.04	
	**+0.93**	**+5.20**	**+2.01**	**−17.53**	
NICS(c) **NICS(c1)_zz_**	−12.60	+3.49	−7.56	+0.90	
	**−35.25**	**−0.98**	**−22.10**	**−6.01**	
NICS(d) **NICS(d1)_zz_**	−13.27	−8.50	+1.51	+2.61	
	**−25.94**	**−24.18**	**−4.54**	**−1.77**	

**Table 5:** Calculated Relative Energies (kcal/mol) and NICS Values (ppm) for Diprotonated Pyrazoloporphyrin Dications.

	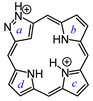 [PzP-2,22,23,24-H]^2+^	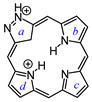 [PzP-2,21,22,24-H]^2+^	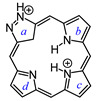 [PzP-2,21,23,24-H]^2+^	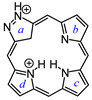 [PzP-2,21,22,23-H]^2+^
ΔG298 (kcal/mol) ΔE (kcal/mol)	0.00	13.22	16.53	16.04
	0.00	15.13	18.53	18.16
NICS(0) **NICS(01)_zz_**	−4.14	−11.63	−10.59	−12.24
	**−7.49**	**−27.10**	**−24.93**	**−29.07**
NICS(a) **NICS(a1)_zz_**	−9.29	−20.24	−18.67	−21.04
	**−16.86**	**−49.56**	**−46.46**	**−51.64**
NICS(b) **NICS(b1)_zz_**	−6.32	−6.31	−7.68	+6.36
	**−20.75**	**−19.11**	**−14.35**	**+6.24**
NICS(c) **NICS(c1)_zz_**	−4.96	+2.65	−8.50	−7.72
	**−10.04**	**−2.31**	**−27.00**	**−25.27**
NICS(d) **NICS(d1)_zz_**	−7.20	−7.85	+2.19	−9.67
	**−22.55**	**−22.77**	**−2.23**	**−18.57**

## Data Availability

Not applicable.

## Supplementary Materials

The following supporting information can be downloaded at: https://www.mdpi.com/article/10.3390/molecules28062854/s1, Table S1: AICD plots for pyrazoloporphyrin tautomers and related protonated species; Table S2: Calculated energies (hartrees) for all the optimized structures. Table S3: Cartesian coordinates.
