# Supplementary material for: Aromatic Character and Relative Stability of Pyrazoloporphyrin Tautomers and Related Protonated Species: Insights into How Pyrazole Changes the Properties of Carbaporphyrinoid Systems
Source: Molecules. 2023 Mar 22;28(6):2854. doi: 10.3390/molecules28062854 (PMC10056226; doi:10.3390/molecules28062854)
Supplement: Supplementary file 1 [file molecules-28-02854-s001.zip › molecules-2269926-supplementary.pdf]

Supplementary Material for
Aromatic Character and Relative Stability of Pyrazoloporphyrin Tautomers and Related Protonated Species: Insights into How Pyrazole Changes the Properties of Carbaporphyrinoid Systems

Deyaa I. AbuSalim and Timothy D. Lash*
Department of Chemistry, Illinois State University, Normal, IL 61790-4160, USA

Table of Contents

Page

S2-S6 **Table S1:** AICD plots of pyrazoloporphyrin tautomers and related protonated species

S7 **Table S2:** Calculated energies for the optimized structures.

S8-S25 **Table S3:** Cartesian coordinates

Table S1: AICD plots for pyrazoloporphyrin tautomers and related protonated species.

Molecule	*Iso 0.05*	*Iso 0.07*
**PzP-22,24-H**	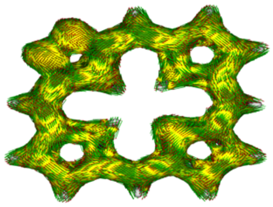	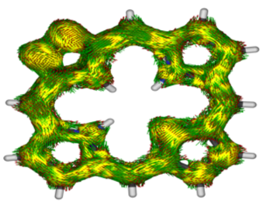
**PzP-22,23-H**	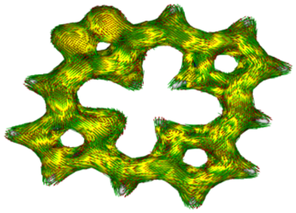	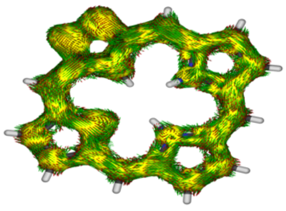
**PzP-2,23-H**	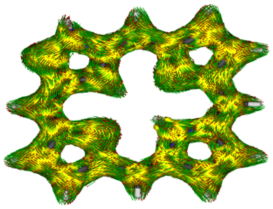	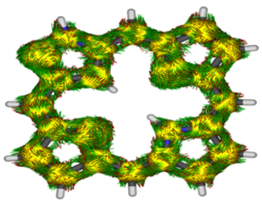
**PzP-2,22-H**	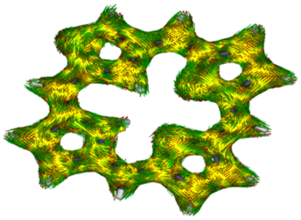	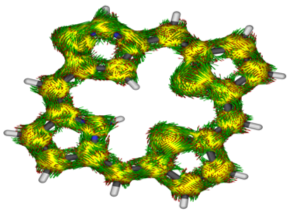
**PzP-2,24-H**	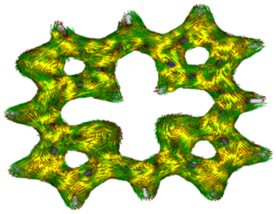	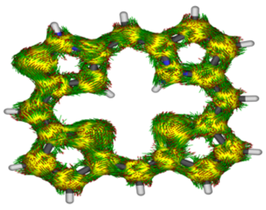
**PzP-2,20-H**	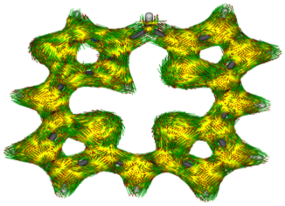	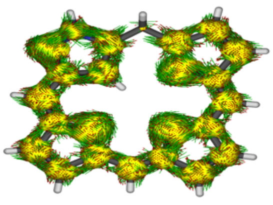

**PzP-2,15-H**	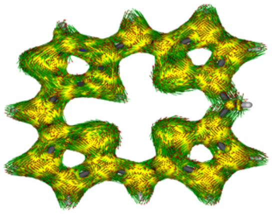	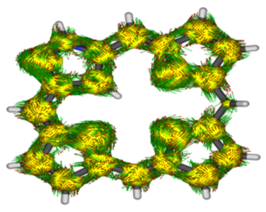
**PzP-2,10-H**	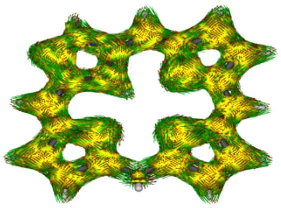	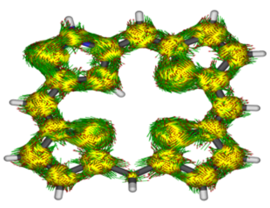
**PzP-2,5-H**	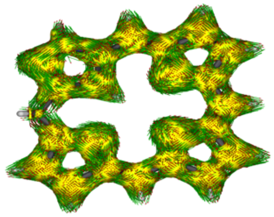	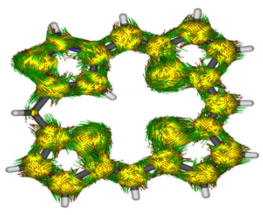
**PzP-5,22-H**	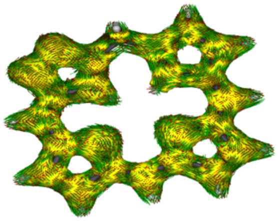	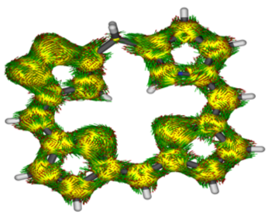
**PzP-5,23-H**	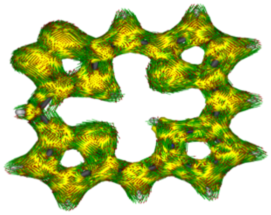	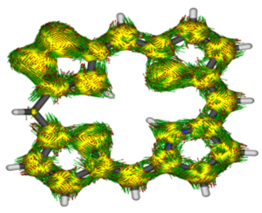
**PzP-5,24-H**	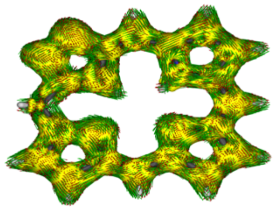	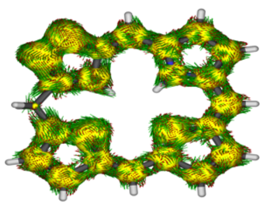

**PzP-10,22-H**	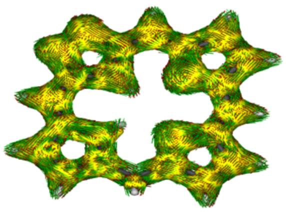	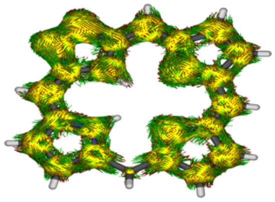
**PzP-10,23-H**	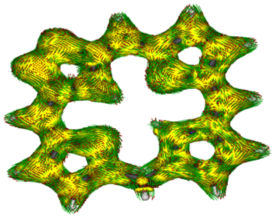	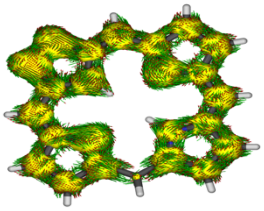
**PzP-10,24-H**	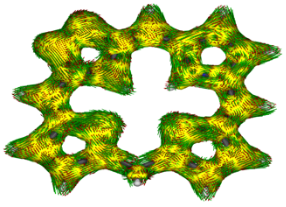	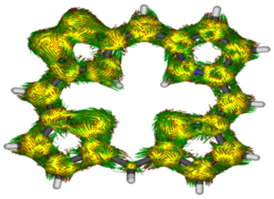
**PzP-21,22-H**	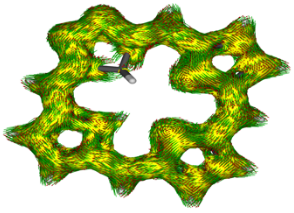	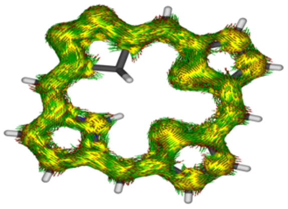
**PzP-21,23-H**	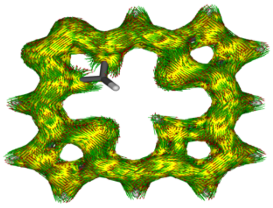	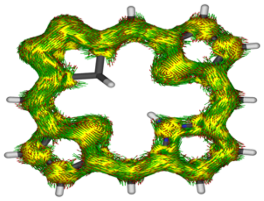
**[PzP-22,23,24-H]+**	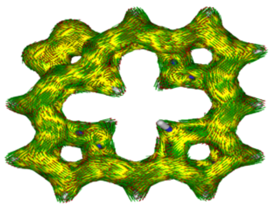	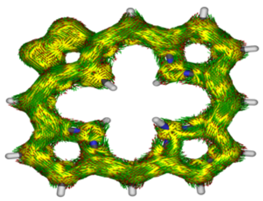

**[PzP-2,22,24-H]+**	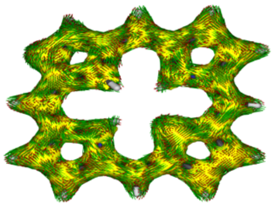	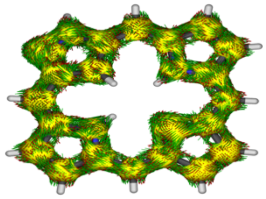
**[PzP-2,22,23-H]+**	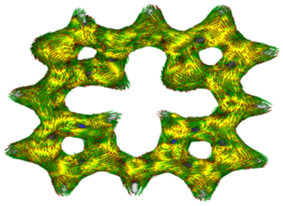	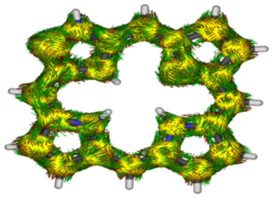
**[PzP-2,23,24-H]+**	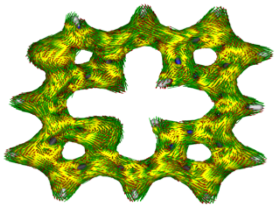	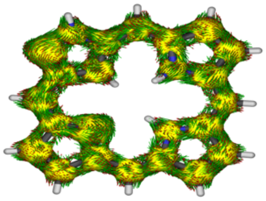
**[PzP-21,22,24-H]+**	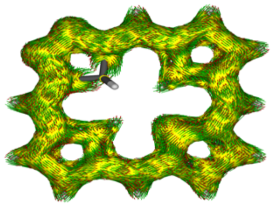	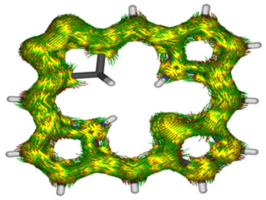
**[PzP-21,22,23-H]+**	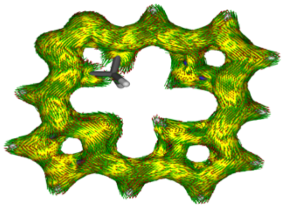	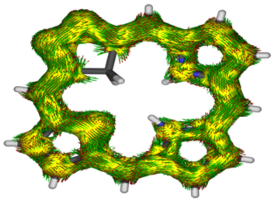
**[PzP-2,21,22-H]+**	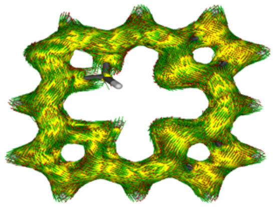	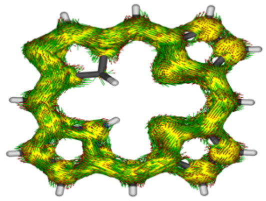

**[PzP-2,21,23-H]+**	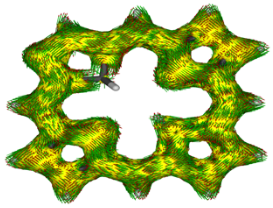	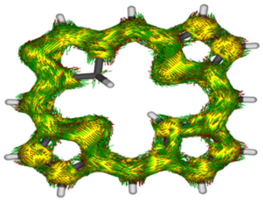
**[PzP-2,21,24-H]+**	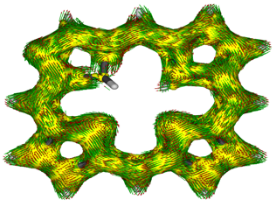	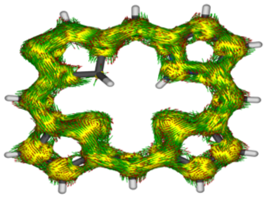
**[PzP-2,22,23,24-H]2+**	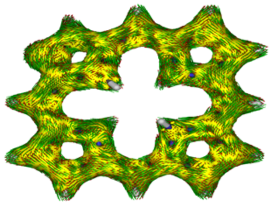	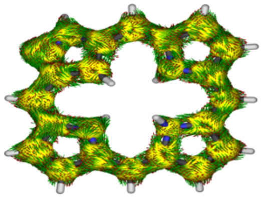
**[PzP-2,21,22,24-H]2+**	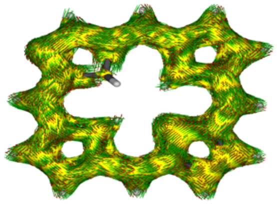	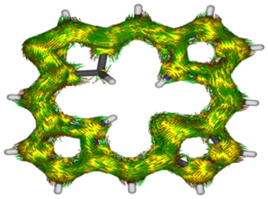
**[PzP-2,21,23,24-H]2+**	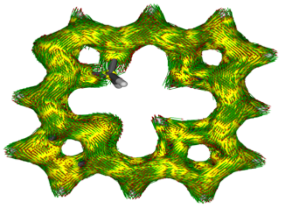	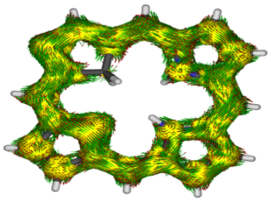
**[PzP-2,21,22,23-H]2+**	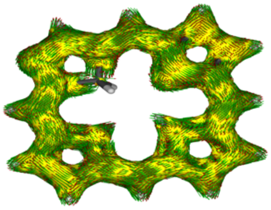	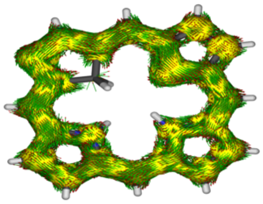

Table S2: Calculated energies for the optimized structures.

Molecule	*E-M06-2X/cc-pVTZ (Hartrees)*	*G298-6-311++G(d,p) (Hartrees)*	*ΔE (kcal/mol)*	*ΔG (kcal/mol)*
**PzP-22,24-H**	-1005.480623	-1005.140597	0.00	0.00
**PzP-22,23-H**	-1005.469997	-1005.130723	6.67	6.20
**PzP-2,23-H**	-1005.480029	-1005.140988	0.37	-0.25
**PzP-2,22-H**	-1005.468069	-1005.129065	7.88	7.24
**PzP-2,24-H**	-1005.469343	-1005.130369	7.08	6.42
**PzP-2,20-H**	-1005.429388	-1005.095753	32.15	28.14
**PzP-2,15-H**	-1005.430438	-1005.096963	31.49	27.38
**PzP-2,10-H**	-1005.430159	-1005.096685	31.67	27.56
**PzP-2,5-H**	-1005.428409	-1005.095289	32.76	28.43
**PzP-5,22-H**	-1005.432601	-1005.097142	30.13	27.27
**PzP-5,23-H**	-1005.445125	-1005.108831	22.28	19.93
**PzP-5,24-H**	-1005.439592	-1005.103574	25.75	23.23
**PzP-10,22-H**	-1005.435956	-1005.101109	28.03	24.78
**PzP-10,23-H**	-1005.442049	-1005.107036	24.21	21.06
**PzP-10,24-H**	-1005.439426	-1005.104000	25.85	22.96
**PzP-21,22-H**	-1005.451427	-1005.114761	18.32	16.21
**PzP-21,23-H**	-1005.462669	-1005.125990	11.27	9.17
**[PzP-22,23,24-H]$^+$**	-1005.856533	-1005.500855	16.97	16.70
**[PzP-2,22,24-H]$^+$**	-1005.883570	-1005.527467	0.00	0.00
**[PzP-2,22,23-H]$^+$**	-1005.877750	-1005.521954	3.65	3.46
**[PzP-2,23,24-H]$^+$**	-1005.876739	-1005.520979	4.29	4.07
**[PzP-21,22,24-H]$^+$**	-1005.850275	-1005.497172	20.89	19.01
**[PzP-21,22,23-H]$^+$**	-1005.842394	-1005.489318	25.84	23.94
**[PzP-2,21,22-H]$^+$**	-1005.841309	-1005.489281	26.52	23.96
**[PzP-2,21,23-H]$^+$**	-1005.856016	-1005.503717	17.29	14.90
**[PzP-2,21,24-H]$^+$**	-1005.843930	-1005.491283	24.87	22.71
**[PzP-2,22,23,24-H]$^{2+}$**	-1006.142287	-1005.771668	0.00	0.00
**[PzP-2,21,22,24-H]$^{2+}$**	-1006.118183	-1005.750606	15.13	13.22
**[PzP-2,21,23,24-H]$^{2+}$**	-1006.112757	-1005.745321	18.53	16.53
**[PzP-2,21,22,23-H]$^{2+}$**	-1006.113341	-1005.746100	18.16	16.04

Table S3. Cartesian Coordinates

PzP-22,24-H

C	-0.672175000	4.144190000	-0.111772000
C	0.673129000	4.144054000	-0.111715000
C	1.084762000	2.741760000	-0.059072000
N	0.000233000	1.916249000	-0.031981000
C	-1.084098000	2.742026000	-0.058849000
C	-4.313332000	-0.714944000	0.077837000
C	-4.312273000	0.653332000	0.070102000
C	-2.948224000	1.089083000	0.029369000
N	-2.178632000	-0.029276000	0.038544000
C	-2.954859000	-1.166709000	0.057921000
C	-2.430871000	2.383359000	-0.027298000
N	0.633667000	-4.045264000	-0.333919000
N	-0.634423000	-4.045147000	-0.333999000
C	-1.100963000	-2.770580000	0.073100000
C	-0.000363000	-1.969329000	0.372443000
C	1.100228000	-2.770762000	0.073468000
C	-2.462752000	-2.462677000	0.025753000
C	4.312552000	0.652287000	0.069506000
C	4.313114000	-0.715953000	0.077787000
C	2.954426000	-1.167318000	0.058645000
N	2.178615000	-0.029604000	0.039064000
C	2.948598000	1.088520000	0.029216000
C	2.431527000	2.382895000	-0.027675000
C	2.462115000	-2.463165000	0.026434000
H	-1.348589000	4.985954000	-0.140549000
H	1.349706000	4.985682000	-0.140459000
H	-5.168182000	-1.373269000	0.108290000
H	-5.164915000	1.314439000	0.083569000
H	-1.179648000	0.030936000	-0.107266000
H	-3.153430000	3.190217000	-0.045954000
H	-0.000569000	-1.029991000	0.897921000
H	-3.167424000	-3.275949000	-0.104956000
H	5.165517000	1.312988000	0.082147000
H	5.167813000	-1.374491000	0.107863000
H	1.179411000	0.031303000	-0.105010000
H	3.154200000	3.189632000	-0.046609000
H	3.166643000	-3.276564000	-0.104162000

PzP-22,23-H

C	0.251710000	4.168899000	-0.160031000
C	1.587802000	3.926842000	-0.120998000
C	1.780988000	2.506457000	-0.029393000
N	0.522300000	1.939277000	-0.022385000
C	-0.448932000	2.909755000	-0.092816000
C	-4.371301000	0.209248000	0.183572000
C	-4.031141000	1.539751000	0.164112000
C	-2.617972000	1.636079000	-0.015166000
N	-2.149807000	0.357152000	-0.077644000
C	-3.182171000	-0.558521000	0.035264000
C	-1.831083000	2.791710000	-0.080455000
N	-0.253949000	-4.104118000	-0.322602000
N	-1.498060000	-3.861072000	-0.321100000
C	-1.705768000	-2.504264000	0.046515000
C	-0.464447000	-1.926791000	0.319163000
C	0.451912000	-2.929409000	0.049037000
C	-2.978723000	-1.933313000	0.014249000
C	4.364549000	-0.312299000	0.067729000
C	4.040259000	-1.626763000	0.049736000
C	2.586749000	-1.693015000	0.084338000
N	2.054801000	-0.433678000	0.118317000
C	3.099041000	0.408034000	0.093702000
C	2.969910000	1.813063000	0.035948000
C	1.848988000	-2.870320000	0.019600000
H	-0.246653000	5.123672000	-0.230933000
H	2.391973000	4.646046000	-0.152523000
H	0.457727000	0.930755000	0.082181000

H	-5.353698000	-0.213708000	0.328325000
H	-4.688227000	2.387876000	0.279307000
H	-1.259710000	0.090506000	-0.471890000
H	-2.373398000	3.728809000	-0.092389000
H	-0.225660000	-0.980962000	0.763207000
H	-3.840035000	-2.586422000	-0.068907000
H	5.345621000	0.139010000	0.043728000
H	4.701099000	-2.480799000	0.017490000
H	3.877320000	2.405166000	0.018313000
H	2.384436000	-3.803732000	-0.122647000

PzP-2,23-H

C	4.349699000	-0.663580000	0.012045000
C	4.320699000	0.683214000	-0.014434000
C	2.898341000	1.057561000	0.011024000
N	2.112529000	0.004133000	0.060759000
C	2.954357000	-1.105405000	0.058754000
N	-0.565984000	-3.986629000	-0.229057000
N	0.753590000	-4.023714000	-0.243146000
C	1.137204000	-2.773047000	0.075300000
C	0.021519000	-1.952247000	0.312988000
C	-1.078467000	-2.748018000	0.080808000
C	2.527707000	-2.396437000	0.058847000
C	-4.332372000	0.620619000	-0.003102000
C	-4.335791000	-0.725177000	0.018617000
C	-2.928596000	-1.138744000	0.050772000
N	-2.109519000	-0.018834000	0.045931000
C	-2.914448000	1.023701000	0.009282000
C	-2.476156000	-2.422783000	0.057180000
C	0.642227000	4.174685000	-0.052949000
C	-0.704315000	4.166735000	-0.052943000
C	-1.155669000	2.781269000	-0.038932000
N	-0.020332000	2.006774000	-0.031849000
C	1.110878000	2.795326000	-0.038397000
C	2.419119000	2.406080000	-0.024745000
C	-2.463213000	2.375698000	-0.025967000
H	5.209173000	-1.318142000	0.000945000
H	5.151656000	1.372511000	-0.056663000
H	-1.080573000	-4.818269000	-0.477060000
H	0.017846000	-0.930019000	0.618986000
H	3.259519000	-3.194976000	0.001116000
H	-5.176556000	1.294212000	-0.034972000
H	-5.183178000	-1.395534000	0.015616000
H	-3.202489000	-3.228228000	0.014104000
H	1.303480000	5.028111000	-0.057201000
H	-1.376012000	5.011868000	-0.057357000
H	-0.015875000	0.992890000	-0.067204000
H	3.157018000	3.199101000	-0.042350000
H	-3.212342000	3.157646000	-0.041305000

PzP-2,22-H

C	3.983896000	-1.735676000	-0.043314000
C	4.354716000	-0.437687000	-0.019215000
C	3.147869000	0.374196000	0.010064000
N	2.095973000	-0.486581000	-0.003402000
C	2.530132000	-1.806897000	-0.034636000
N	-1.626645000	-3.699974000	0.204682000
N	-0.356314000	-4.059096000	0.223396000
C	0.328200000	-2.933583000	-0.060654000
C	-0.551947000	-1.858373000	-0.277432000
C	-1.819295000	-2.370238000	-0.071912000
C	1.768868000	-2.929908000	-0.051610000
C	-4.003461000	1.739528000	0.015946000
C	-4.420899000	0.458659000	0.000894000
C	-3.202948000	-0.358835000	-0.055767000
N	-2.087509000	0.459541000	-0.077719000
C	-2.525347000	1.691549000	-0.028102000
C	-3.099680000	-1.716854000	-0.048591000

C	1.711072000	3.912919000	0.063993000
C	0.400741000	4.196972000	0.053115000
C	-0.311165000	2.904153000	0.020548000
N	0.600452000	1.868687000	0.014786000
C	1.804590000	2.440691000	0.039595000
C	3.038234000	1.748668000	0.039037000
C	-1.679673000	2.849072000	0.001576000
H	4.621814000	-2.606408000	-0.073444000
H	5.354949000	-0.032264000	-0.021884000
H	1.151367000	-0.118685000	0.084892000
H	-2.331283000	-4.388779000	0.422097000
H	-0.370627000	-0.840621000	-0.562320000
H	2.273078000	-3.888165000	-0.027142000
H	-4.603934000	2.636840000	0.063730000
H	-5.433095000	0.081980000	0.027315000
H	-3.999731000	-2.320559000	0.009041000
H	2.551975000	4.590844000	0.086557000
H	-0.080940000	5.164228000	0.063235000
H	3.952512000	2.327191000	0.054183000
H	-2.191183000	3.806224000	0.020329000

PzP-2,24-H

C	-4.428656000	0.401400000	0.008565000
C	-4.024224000	1.687978000	0.030185000
C	-2.550394000	1.654628000	-0.031966000
N	-2.097543000	0.430722000	-0.099536000
C	-3.207124000	-0.403419000	-0.068028000
N	-0.415684000	-3.982325000	0.226015000
N	-1.713766000	-3.729494000	0.234980000
C	-1.811475000	-2.425910000	-0.069406000
C	-0.536823000	-1.863884000	-0.295947000
C	0.360144000	-2.886445000	-0.072507000
C	-3.090870000	-1.758436000	-0.052453000
C	4.360598000	-0.384212000	-0.020492000
C	4.004938000	-1.685174000	-0.050223000
C	2.549832000	-1.770596000	-0.036804000
N	2.100024000	-0.462994000	0.002259000
C	3.144846000	0.416656000	0.014288000
C	1.795929000	-2.901496000	-0.059929000
C	0.345683000	4.202371000	0.055065000
C	1.660748000	3.934341000	0.067042000
C	1.771494000	2.465395000	0.043247000
N	0.581587000	1.877072000	0.016881000
C	-0.349052000	2.903532000	0.020880000
C	-1.712487000	2.824539000	-0.000492000
C	3.020189000	1.782098000	0.042856000
H	-5.436202000	0.013567000	0.045978000
H	-4.633381000	2.578477000	0.092970000
H	-0.095779000	-4.907848000	0.468074000
H	-0.369475000	-0.849163000	-0.599468000
H	-3.976802000	-2.379549000	0.023707000
H	5.356727000	0.031460000	-0.023129000
H	4.652986000	-2.548131000	-0.086509000
H	1.148774000	-0.112530000	0.090464000
H	2.316494000	-3.851801000	-0.051616000
H	-0.147299000	5.163898000	0.064864000
H	2.492522000	4.623362000	0.090616000
H	-2.237755000	3.774715000	0.020523000
H	3.927099000	2.372455000	0.056047000

PzP-2,20-H

C	-4.374536000	-0.889040000	-0.000046000
C	-4.398903000	0.462636000	-0.000039000
C	-2.991124000	0.891078000	-0.000016000
N	-2.172502000	-0.125414000	-0.000004000
C	-2.961356000	-1.262693000	-0.000025000
N	0.755598000	-3.941380000	0.000056000
N	-0.566063000	-4.043109000	0.000040000

C	-0.996496000	-2.774050000	0.000000000
C	0.091206000	-1.858141000	-0.000016000
C	1.211130000	-2.646878000	0.000024000
C	-2.412225000	-2.509513000	-0.000018000
C	4.308249000	0.969007000	-0.000071000
C	4.419759000	-0.372480000	-0.000070000
C	3.031535000	-0.888364000	0.000008000
N	2.153850000	0.056044000	0.000055000
C	2.865438000	1.258703000	-0.000005000
C	2.679302000	-2.347144000	0.000030000
C	-0.925067000	4.167690000	0.000065000
C	0.418802000	4.225228000	0.000063000
C	0.880330000	2.822537000	0.000004000
N	-0.108607000	1.968861000	-0.000032000
C	-1.269486000	2.739705000	0.000008000
C	-2.546176000	2.268357000	0.000007000
C	2.296729000	2.484810000	-0.000024000
H	-5.209391000	-1.574506000	-0.000064000
H	-5.261023000	1.114363000	-0.000051000
H	1.305770000	-4.786916000	0.000082000
H	0.041369000	-0.784249000	-0.000038000
H	-3.063356000	-3.378599000	-0.000024000
H	5.095665000	1.708726000	-0.000120000
H	5.320792000	-0.969593000	-0.000117000
H	3.156268000	-2.808332000	-0.874750000
H	3.156256000	-2.808308000	0.874829000
H	-1.635680000	4.981514000	0.000104000
H	1.054693000	5.099125000	0.000097000
H	-3.333324000	3.017927000	0.000033000
H	2.973352000	3.335129000	-0.000064000

PzP-2,15-H

C	4.440861000	-0.555698000	0.000023000
C	4.347538000	0.791628000	0.000033000
C	2.905378000	1.098295000	0.000005000
N	2.179391000	0.019254000	-0.000010000
C	3.064173000	-1.052184000	0.000007000
N	-0.441078000	-4.001037000	-0.000024000
N	0.878708000	-4.004609000	-0.000024000
C	1.225837000	-2.704955000	-0.000005000
C	0.085804000	-1.870932000	-0.000001000
C	-0.986799000	-2.738585000	-0.000013000
C	2.623733000	-2.336714000	0.000007000
C	-4.386986000	0.467507000	0.000016000
C	-4.365899000	-0.880355000	0.000022000
C	-2.950293000	-1.257801000	-0.000005000
N	-2.157041000	-0.102556000	-0.000009000
C	-2.969416000	0.897505000	0.000022000
C	-2.410391000	-2.497463000	-0.000008000
C	0.594229000	4.206308000	-0.000030000
C	-0.747925000	4.168224000	-0.000055000
C	-1.110946000	2.730725000	0.000014000
N	-0.078833000	1.951439000	0.000015000
C	1.042043000	2.805565000	-0.000006000
C	2.345416000	2.439290000	-0.000007000
C	-2.561073000	2.337026000	0.000032000
H	5.332155000	-1.165926000	0.000034000
H	5.150176000	1.515415000	0.000051000
H	-0.931573000	-4.882569000	-0.000038000
H	0.050124000	-0.795195000	0.000009000
H	3.340413000	-3.151922000	0.000015000
H	-5.248599000	1.120530000	0.000024000
H	-5.202244000	-1.564182000	0.000031000
H	-3.076080000	-3.356104000	-0.000004000
H	1.246163000	5.067935000	-0.000049000
H	-1.444572000	4.995118000	-0.000089000
H	3.069197000	3.249851000	-0.000022000
H	-3.032427000	2.822638000	0.866047000

H	-3.032458000	2.822660000	-0.865954000

PzP-2,10-H

C	4.370526000	-0.860045000	0.000063000
C	4.381094000	0.488671000	0.000087000
C	2.962479000	0.905807000	0.000014000
N	2.156916000	-0.099916000	-0.000040000
C	2.959928000	-1.251684000	-0.000007000
N	-0.733287000	-3.966753000	-0.000042000
N	0.584307000	-4.053650000	-0.000047000
C	1.016404000	-2.780617000	-0.000026000
C	-0.063663000	-1.874923000	-0.000006000
C	-1.194802000	-2.671020000	-0.000021000
C	2.438540000	-2.498012000	-0.000008000
C	-4.351316000	0.772003000	0.000075000
C	-4.433747000	-0.575250000	0.000065000
C	-3.050758000	-1.057868000	0.000001000
N	-2.175681000	0.017643000	-0.000037000
C	-2.909424000	1.091305000	0.000012000
C	-2.594591000	-2.338296000	0.000002000
C	0.728156000	4.171415000	-0.000065000
C	-0.612967000	4.208587000	-0.000055000
C	-1.058745000	2.805782000	-0.000001000
N	0.062677000	1.952589000	0.000031000
C	1.094245000	2.732053000	-0.000013000
C	2.544510000	2.343575000	0.000001000
C	-2.360939000	2.436179000	-0.000003000
H	5.211766000	-1.537825000	0.000098000
H	5.237336000	1.148860000	0.000144000
H	-1.278470000	-4.815468000	-0.000058000
H	-0.023007000	-0.799246000	0.000011000
H	3.103068000	-3.356104000	0.000017000
H	-5.159867000	1.489112000	0.000121000
H	-5.320330000	-1.192394000	0.000104000
H	-3.316700000	-3.150231000	0.000030000
H	1.424229000	4.998805000	-0.000104000
H	-1.266080000	5.069281000	-0.000088000
H	3.011750000	2.833846000	-0.865774000
H	3.011737000	2.833861000	0.865774000
H	-3.089523000	3.242139000	-0.000019000

PzP-2,5-H

C	4.418761000	-0.365430000	-0.000260000
C	4.303948000	0.974074000	-0.000287000
C	2.858117000	1.259201000	-0.000063000
N	2.150305000	0.054978000	0.000137000
C	3.030130000	-0.889910000	-0.000003000
N	-0.439032000	-3.997504000	0.000184000
N	0.886442000	-3.991780000	0.000253000
C	1.235557000	-2.699236000	0.000104000
C	0.105365000	-1.868171000	-0.000041000
C	-0.979364000	-2.739139000	0.000026000
C	2.696567000	-2.348848000	0.000095000
C	-4.397002000	0.458428000	-0.000244000
C	-4.366731000	-0.892526000	-0.000273000
C	-2.950142000	-1.257220000	-0.000063000
N	-2.167122000	-0.118945000	0.000091000
C	-2.989432000	0.894286000	-0.000034000
C	-2.392279000	-2.501345000	-0.000067000
C	0.413700000	4.227884000	0.000258000
C	-0.929164000	4.173022000	0.000274000
C	-1.275816000	2.743859000	0.000064000
N	-0.115828000	1.971220000	-0.000108000
C	0.874385000	2.822283000	0.000010000
C	2.290618000	2.485707000	-0.000108000
C	-2.552239000	2.274057000	0.000066000
H	5.320518000	-0.961404000	-0.000401000
H	5.088288000	1.717065000	-0.000467000

H	-0.925170000	-4.880652000	0.000307000
H	0.064223000	-0.793345000	-0.000141000
H	3.178285000	-2.810122000	-0.870510000
H	3.178261000	-2.810010000	0.870776000
H	-5.262336000	1.105799000	-0.000364000
H	-5.199268000	-1.580908000	-0.000427000
H	-3.054196000	-3.363686000	-0.000165000
H	1.051212000	5.100612000	0.000392000
H	-1.638478000	4.987952000	0.000437000
H	2.966861000	3.336062000	-0.000239000
H	-3.341440000	3.020998000	0.000165000

PzP-5,22-H

C	-1.257772000	4.081357000	-0.121051000
C	0.092302000	4.212254000	-0.093171000
C	0.629787000	2.861151000	-0.054968000
N	-0.337257000	1.944747000	-0.065621000
C	-1.510332000	2.656623000	-0.101295000
C	-4.122155000	-1.273099000	0.211845000
C	-4.328436000	0.097604000	0.150455000
C	-3.073672000	0.708376000	-0.033246000
N	-2.140190000	-0.309637000	-0.099259000
C	-2.744932000	-1.503542000	0.047138000
C	-2.765961000	2.072412000	-0.073242000
N	1.309858000	-3.937292000	-0.299662000
N	0.058976000	-4.079645000	-0.354241000
C	-0.590321000	-2.868312000	0.059240000
C	0.335435000	-1.949264000	0.386856000
C	1.589819000	-2.600574000	0.098387000
C	-2.076189000	-2.839946000	0.002157000
C	4.186916000	1.299495000	0.110215000
C	4.428124000	-0.030857000	0.101372000
C	3.110068000	-0.669248000	0.058126000
N	2.133654000	0.208367000	0.030818000
C	2.731726000	1.447612000	0.059079000
C	2.045173000	2.627356000	0.010212000
C	2.836383000	-2.089270000	-0.021805000
H	-2.010087000	4.855945000	-0.149603000
H	0.677768000	5.120264000	-0.092050000
H	-4.861293000	-2.045690000	0.354629000
H	-5.263778000	0.628141000	0.243947000
H	-1.153129000	-0.108186000	-0.247292000
H	-3.620764000	2.741921000	-0.055415000
H	0.250118000	-0. 945185000	0.76139900
H	-2.376982000	-3.349472000	-0.921759000
H	-2.489654000	-3.452367000	0.812047000
H	4.900726000	2.109840000	0.141057000
H	5.379889000	-0.540981000	0.126395000
H	2.649492000	3.530844000	0.024311000
H	3.646627000	-2.771630000	-0.259724000

PzP-5,23-H

C	0.412720000	4.180975000	-0.000033000
C	-0.972512000	4.097886000	0.000023000
C	-1.318138000	2.734130000	-0.000067000
N	-0.148726000	2.019885000	-0.000191000
C	0.916896000	2.868279000	-0.000154000
C	4.369281000	-0.389470000	0.000088000
C	4.284913000	0.956444000	-0.000064000
C	2.857956000	1.269073000	-0.000083000
N	2.116566000	0.083347000	0.000035000
C	2.983840000	-0.888448000	0.000127000
C	2.305026000	2.507630000	-0.000176000
N	-0.436833000	-4.180595000	-0.000505000
N	0.820799000	-4.099295000	-0.000503000
C	1.222179000	-2.715471000	0.000238000
C	0.130626000	-1.939978000	0.000765000
C	-0.972924000	-2.853724000	0.000151000

C	2.662363000	-2.353346000	0.000214000
C	-4.392553000	0.391605000	0.000083000
C	-4.288860000	-0.958685000	0.000061000
C	-2.858111000	-1.254758000	0.000033000
N	-2.131307000	-0.153540000	0.000021000
C	-3.026780000	0.894868000	0.000050000
C	-2.637589000	2.208308000	0.000002000
C	-2.298369000	-2.594739000	-0.000101000
H	1.020200000	5.072568000	0.000026000
H	-1.681909000	4.911050000	0.000134000
H	-0.090388000	1.008130000	-0.000349000
H	5.256740000	-1.006136000	0.000152000
H	5.085237000	1.681866000	-0.000146000
H	2.984009000	3.354553000	-0.000230000
H	0.072161000	-0.879627000	0.001427000
H	3.154082000	-2.812643000	0.868530000
H	3.153999000	-2.812691000	-0.868123000
H	-5.287034000	0.997004000	0.000104000
H	-5.082146000	-1.691918000	0.000065000
H	-3.421177000	2.959874000	0.000037000
H	-2.987047000	-3.434097000	-0.000569000

PzP-5,24-H

C	-0.499875000	4.175829000	-0.053079000
C	-1.817439000	3.884134000	-0.013051000
C	-1.900544000	2.431298000	-0.001929000
N	-0.628358000	1.871612000	-0.031245000
C	0.205897000	2.891259000	-0.059908000
C	4.380193000	0.564055000	0.000995000
C	3.958176000	1.839531000	-0.075738000
C	2.489041000	1.784614000	-0.037857000
N	2.077832000	0.454687000	0.063496000
C	3.155772000	-0.262620000	0.081409000
C	1.656493000	2.848817000	-0.091999000
N	0.475290000	-4.098635000	-0.272286000
N	1.694641000	-3.766376000	-0.242585000
C	1.814797000	-2.394681000	0.134471000
C	0.589913000	-1.877172000	0.355373000
C	-0.307180000	-2.956933000	0.052179000
C	3.160270000	-1.758019000	0.172602000
C	-4.332712000	-0.505027000	0.078723000
C	-3.924933000	-1.834391000	0.031397000
C	-2.520841000	-1.847981000	-0.029210000
N	-2.101640000	-0.549789000	-0.030988000
C	-3.174234000	0.289366000	0.030627000
C	-3.060157000	1.706125000	0.029816000
C	-1.654607000	-2.985402000	-0.094143000
H	-0.028382000	5.147696000	-0.072780000
H	-2.659093000	4.560835000	0.005381000
H	5.394451000	0.191017000	-0.000812000
H	4.548748000	2.740594000	-0.156022000
H	2.129666000	3.824246000	-0.162235000
H	0.381398000	-0.875869000	0.680961000
H	3.763428000	-2.164926000	-0.647871000
H	3.696702000	-2.046603000	1.087126000
H	-5.341513000	-0.127436000	0.140195000
H	-4.547024000	-2.715515000	0.053123000
H	-1.157951000	-0.183293000	-0.141254000
H	-3.995925000	2.254553000	0.059130000
H	-2.117029000	-3.942605000	-0.313755000

PzP-10,22-H

C	-1.671871000	-3.904356000	0.041000000
C	-0.353490000	-4.181067000	0.032045000
C	0.347417000	-2.898157000	0.019448000
N	-0.601442000	-1.855275000	0.021854000
C	-1.764441000	-2.442566000	0.032901000
C	-3.910838000	1.848911000	-0.064599000

C	-4.331497000	0.524401000	-0.079514000
C	-3.178931000	-0.272880000	-0.006037000
N	-2.107000000	0.532094000	0.036981000
C	-2.505198000	1.860138000	-0.001218000
C	-3.101481000	-1.762489000	0.051398000
N	1.720585000	3.806862000	0.169065000
N	0.489328000	4.121622000	0.172537000
C	-0.278245000	2.955859000	-0.052429000
C	0.597649000	1.870544000	-0.236582000
C	1.847499000	2.417019000	-0.063342000
C	-1.654360000	2.976788000	0.011549000
C	4.034556000	-1.712749000	-0.017999000
C	4.442750000	-0.419440000	-0.015328000
C	3.228766000	0.384195000	-0.023471000
N	2.121390000	-0.437522000	-0.015980000
C	2.571743000	-1.667816000	-0.014928000
C	1.705363000	-2.820816000	0.004555000
C	3.104695000	1.753629000	-0.029039000
H	-2.506897000	-4.589869000	0.050849000
H	0.130797000	-5.146868000	0.031622000
H	-4.526304000	2.734502000	-0.110423000
H	-5.343726000	0.155175000	-0.128889000
H	-1.169625000	0.143988000	0.130596000
H	-3.624649000	-2.099276000	0.955959000
H	-3.691464000	-2.167279000	-0.779640000
H	0.413175000	0.842082000	-0.482855000
H	-2.131469000	3.948223000	0.106450000
H	4.644390000	-2.604639000	-0.011875000
H	5.453025000	-0.037210000	-0.016410000
H	2.216560000	-3.780183000	0.005371000
H	3.995649000	2.371994000	0.027573000

PzP-10,23-H

C	-0.924925000	-4.077642000	-0.068934000
C	0.458618000	-4.145052000	-0.129966000
C	0.960692000	-2.835369000	-0.016723000
N	-0.137436000	-1.998870000	0.119028000
C	-1.270897000	-2.726718000	0.090708000
C	-4.254926000	1.064360000	-0.094566000
C	-4.363193000	-0.278982000	0.005806000
C	-2.989023000	-0.781051000	0.071456000
N	-2.101932000	0.164613000	0.011659000
C	-2.830156000	1.364201000	-0.097148000
C	-2.668885000	-2.227748000	0.274856000
N	0.931951000	4.071895000	0.214077000
N	-0.328175000	4.133590000	0.174978000
C	-0.860396000	2.845521000	-0.151142000
C	0.159696000	1.973288000	-0.353255000
C	1.329404000	2.732822000	-0.067314000
C	-2.270682000	2.601425000	-0.153048000
C	4.297894000	-0.906176000	-0.042317000
C	4.379125000	0.449792000	0.015938000
C	3.009757000	0.929216000	0.049832000
N	2.135870000	-0.072998000	0.040140000
C	2.883215000	-1.216562000	-0.020137000
C	2.316403000	-2.475834000	-0.055635000
C	2.611090000	2.311121000	0.098052000
H	-1.629768000	-4.892200000	-0.126659000
H	1.071323000	-5.025154000	-0.253616000
H	-0.097443000	-0.987078000	0.218874000
H	-5.044240000	1.798220000	-0.168368000
H	-5.260326000	-0.879961000	0.043467000
H	-2.979379000	-2.481288000	1.298967000
H	-3.318569000	-2.826016000	-0.371774000
H	0.111044000	0.949638000	-0.659275000
H	-2.925609000	3.467677000	-0.153738000
H	5.103475000	-1.624378000	-0.088118000
H	5.264661000	1.068140000	0.020189000

H	2.997968000	-3.318164000	-0.133297000
H	3.368061000	3.059283000	0.315406000

PzP-10,24-H

C	0.190037000	4.193342000	-0.037130000
C	1.521005000	3.993426000	-0.028072000
C	1.707738000	2.547169000	-0.021633000
N	0.455339000	1.901405000	-0.027717000
C	-0.430371000	2.855874000	-0.035239000
C	-4.440132000	0.081119000	0.012776000
C	-4.138560000	1.397517000	-0.010565000
C	-2.666408000	1.472723000	-0.000520000
N	-2.114782000	0.305517000	0.031810000
C	-3.163812000	-0.626728000	0.046280000
C	-1.928416000	2.770619000	-0.043855000
N	-0.164785000	-4.155753000	-0.195702000
N	-1.407889000	-3.919223000	-0.187996000
C	-1.628469000	-2.540564000	0.092769000
C	-0.431714000	-1.921956000	0.292008000
C	0.529765000	-2.944030000	0.065546000
C	-2.945188000	-1.966986000	0.067317000
C	4.357351000	-0.193279000	0.066053000
C	4.055751000	-1.551676000	0.041838000
C	2.657039000	-1.680241000	-0.015844000
N	2.138739000	-0.411679000	-0.039309000
C	3.139396000	0.503565000	0.007992000
C	2.909912000	1.916603000	-0.004901000
C	1.888182000	-2.875468000	-0.043008000
H	-0.348504000	5.130470000	-0.042692000
H	2.315455000	4.725273000	-0.024909000
H	-5.415453000	-0.383281000	0.012586000
H	-4.817774000	2.237773000	-0.043513000
H	-2.269259000	3.320850000	-0.932807000
H	-2.283158000	3.386851000	0.794431000
H	-0.317804000	-0.892171000	0.574625000
H	-3.788244000	-2.649222000	0.023869000
H	5.332987000	0.263864000	0.118503000
H	4.746329000	-2.379958000	0.078319000
H	1.170977000	-0.119349000	-0.150124000
H	3.800705000	2.535676000	0.007436000
H	2.428742000	-3.804820000	-0.194196000

PzP-21,22-H

C	-1.535437000	3.959626000	0.000053000
C	-0.200690000	4.169182000	0.000024000
C	0.426041000	2.847709000	0.000015000
N	-0.496223000	1.875000000	0.000006000
C	-1.705991000	2.514904000	0.000032000
C	-4.029908000	-1.585410000	-0.000077000
C	-4.356034000	-0.248454000	-0.000069000
C	-3.141647000	0.487975000	-0.000004000
N	-2.119252000	-0.420472000	0.000036000
C	-2.611304000	-1.694024000	-0.000019000
C	-2.936862000	1.872333000	0.000028000
N	1.513152000	-3.834496000	0.000064000
N	0.248557000	-4.083879000	0.000042000
C	-0.482347000	-2.911094000	0.000010000
C	0.446901000	-1.755858000	-0.000023000
C	1.748058000	-2.473047000	0.000027000
C	-1.846324000	-2.885438000	-0.000002000
C	4.086366000	1.546554000	-0.000037000
C	4.429337000	0.240699000	-0.000081000
C	3.163092000	-0.504327000	0.000001000
N	2.118402000	0.317275000	-0.000012000
C	2.617179000	1.581124000	-0.000024000
C	1.841626000	2.719747000	-0.000010000
C	2.997116000	-1.918757000	0.000034000
H	-2.334208000	4.686720000	0.000073000

H	0.331132000	5.109678000	0.000020000
H	-4.702011000	-2.429479000	-0.000122000
H	-5.341895000	0.190321000	-0.000106000
H	-1.156691000	-0.083383000	0.000103000
H	-3.826947000	2.491817000	0.000032000
H	0.355212000	-1.100316000	0.872211000
H	0.355248000	-1.100392000	-0.872316000
H	-2.383952000	-3.826533000	0.000015000
H	4.736310000	2.409657000	-0.000044000
H	5.418577000	-0.193259000	-0.000118000
H	2.380210000	3.663156000	-0.000022000
H	3.861534000	-2.572654000	0.000063000

<u>PzP-21,23-H</u>

C	0.688187000	-4.114121000	-0.000058000
C	-0.686823000	-4.114317000	-0.000068000
C	-1.120957000	-2.763195000	0.000021000
N	0.000371000	-1.978705000	0.000158000
C	1.121925000	-2.762874000	0.000017000
C	4.338454000	0.710174000	-0.000076000
C	4.341931000	-0.641291000	0.000055000
C	2.937707000	-1.043917000	0.000015000
N	2.123041000	0.052399000	0.000016000
C	2.930857000	1.113495000	-0.000019000
C	2.467012000	-2.337947000	0.000016000
N	-0.643314000	4.055865000	0.000039000
N	0.641946000	4.056035000	0.000038000
C	1.134449000	2.758785000	-0.000017000
C	-0.000358000	1.808864000	-0.000103000
C	-1.135426000	2.758470000	-0.000010000
C	2.463845000	2.460887000	-0.000008000
C	-4.341682000	-0.642670000	-0.000013000
C	-4.338700000	0.708794000	-0.000001000
C	-2.931250000	1.112617000	-0.000025000
N	-2.123042000	0.051819000	-0.000009000
C	-2.937312000	-1.044797000	0.000010000
C	-2.466178000	-2.338671000	0.000019000
C	-2.464722000	2.460165000	0.000002000
H	1.351385000	-4.965400000	-0.000131000
H	-1.349776000	-4.965787000	-0.000148000
H	0.000218000	-0.961833000	0.000289000
H	5.182617000	1.384210000	-0.000129000
H	5.188744000	-1.311945000	0.000098000
H	3.199230000	-3.138391000	0.000012000
H	-0.000264000	1.145932000	0.868323000
H	-0.000271000	1.146103000	-0.868655000
H	3.182178000	3.272966000	0.000039000
H	-5.188251000	-1.313631000	-0.000012000
H	-5.183107000	1.382524000	0.000011000
H	-3.198139000	-3.139350000	0.000003000
H	-3.183325000	3.272006000	0.000057000

<u>[PzP-22,23,24-H]+</u>

C	-0.679901000	4.093310000	-0.435913000
C	0.679931000	4.093163000	-0.436288000
C	1.138830000	2.771494000	-0.124517000
N	-0.000013000	1.973090000	0.015582000
C	-1.138830000	2.771641000	-0.124183000
C	-4.293644000	-0.733411000	0.382229000
C	-4.277833000	0.639458000	0.410495000
C	-2.945398000	1.069836000	0.146139000
N	-2.182714000	-0.055282000	0.010644000
C	-2.970156000	-1.188922000	0.120478000
C	-2.455745000	2.377941000	0.003614000
N	0.630634000	-4.021637000	-0.534881000
N	-0.630686000	-4.021641000	-0.534803000
C	-1.102484000	-2.755710000	-0.087422000
C	0.000029000	-1.956703000	0.215743000

C	1.102382000	-2.755733000	-0.087420000
C	-2.468202000	-2.477522000	-0.020723000
C	4.277878000	0.639371000	0.410629000
C	4.293612000	-0.733406000	0.382523000
C	2.970038000	-1.188944000	0.120597000
N	2.182806000	-0.055260000	0.010352000
C	2.945413000	1.069812000	0.145967000
C	2.455849000	2.377813000	0.003104000
C	2.468198000	-2.477477000	-0.020564000
H	-1.337210000	4.921135000	-0.655917000
H	1.337309000	4.920868000	-0.656542000
H	-0.000042000	1.215150000	0.688640000
H	-5.138090000	-1.382332000	0.559197000
H	-5.107852000	1.301425000	0.604107000
H	-1.312200000	-0.067817000	-0.503079000
H	-3.195069000	3.168882000	-0.025613000
H	0.000078000	-1.043294000	0.782576000
H	-3.166384000	-3.302549000	-0.111805000
H	5.107734000	1.301443000	0.604571000
H	5.137822000	-1.382505000	0.559982000
H	1.312402000	-0.067727000	-0.503574000
H	3.195071000	3.168833000	-0.026332000
H	3.166439000	-3.302478000	-0.111407000

[PzP-2,22,24-H]+

C	-0.716880000	4.163871000	-0.167397000
C	0.620019000	4.175588000	-0.166426000
C	1.045347000	2.764613000	-0.092914000
N	-0.019356000	1.928244000	-0.058135000
C	-1.116738000	2.746864000	-0.093264000
C	-4.320127000	-0.722424000	0.148238000
C	-4.316855000	0.633348000	0.114331000
C	-2.940980000	1.067553000	0.041907000
N	-2.167409000	-0.030832000	0.060519000
C	-2.949111000	-1.183308000	0.110104000
C	-2.448825000	2.379229000	-0.045944000
N	0.634530000	-3.900977000	-0.442136000
N	-0.676254000	-3.956061000	-0.457931000
C	-1.085076000	-2.784941000	0.060381000
C	0.022489000	-1.993684000	0.432196000
C	1.135875000	-2.729377000	0.065082000
C	-2.483632000	-2.459079000	0.083972000
C	4.302684000	0.727400000	0.122355000
C	4.333974000	-0.623804000	0.158630000
C	2.967916000	-1.115538000	0.103998000
N	2.164470000	0.014829000	0.044559000
C	2.914045000	1.139573000	0.036766000
C	2.400594000	2.426635000	-0.045795000
C	2.531923000	-2.401970000	0.088148000
H	-1.404533000	4.995632000	-0.207382000
H	1.292539000	5.019695000	-0.205530000
H	-5.174110000	-1.380853000	0.204121000
H	-5.164930000	1.300242000	0.129091000
H	-1.167667000	0.037658000	-0.084135000
H	-3.186998000	3.170570000	-0.070738000
H	1.160353000	-4.671982000	-0.834061000
H	0.019191000	-1.097479000	1.030299000
H	-3.197897000	-3.272173000	0.028103000
H	5.137184000	1.411241000	0.146337000
H	5.200890000	-1.263823000	0.226638000
H	1.166392000	0.057294000	-0.115620000
H	3.116577000	3.237964000	-0.064047000
H	3.271301000	-3.193687000	0.062592000

[PzP-2,22,23-H]+

C	-0.179630000	4.195796000	-0.225642000
C	-1.510228000	3.982176000	-0.177825000
C	-1.733037000	2.554328000	-0.057532000

N	-0.476736000	1.962837000	-0.046073000
C	0.499701000	2.911835000	-0.132977000
C	4.377733000	0.176083000	0.258780000
C	4.051457000	1.492405000	0.214895000
C	2.627164000	1.597941000	-0.005597000
N	2.140795000	0.336834000	-0.062313000
C	3.172952000	-0.599758000	0.086529000
C	1.879088000	2.774339000	-0.109356000
N	0.190802000	-3.957513000	-0.437144000
N	1.489663000	-3.773600000	-0.450985000
C	1.672602000	-2.532100000	0.036735000
C	0.436673000	-1.948869000	0.382859000
C	-0.518277000	-2.881753000	0.036572000
C	2.985780000	-1.944005000	0.085524000
C	-4.374755000	-0.208663000	0.129688000
C	-4.100274000	-1.525816000	0.136019000
C	-2.640736000	-1.642693000	0.128113000
N	-2.062781000	-0.374766000	0.123993000
C	-3.075059000	0.471786000	0.108937000
C	-2.920407000	1.897118000	0.028433000
C	-1.950707000	-2.813208000	0.063438000
H	0.340824000	5.136941000	-0.316818000
H	-2.302453000	4.714289000	-0.221243000
H	-0.420864000	0.958504000	0.091239000
H	5.352523000	-0.254554000	0.433243000
H	4.707389000	2.340798000	0.335403000
H	1.263429000	0.082857000	-0.494530000
H	2.443773000	3.696999000	-0.130474000
H	-0.181955000	-4.825832000	-0.799925000
H	0.239454000	-1.035292000	0.910776000
H	3.849701000	-2.597989000	0.101545000
H	-5.338516000	0.278504000	0.122412000
H	-4.790101000	-2.356879000	0.142897000
H	-3.819874000	2.500707000	0.013984000
H	-2.515774000	-3.737241000	-0.006408000

[PzP-2,23,24-H]+

C	-1.553108000	3.964938000	0.178330000
C	-0.222482000	4.193701000	0.224930000
C	0.466613000	2.919548000	0.130683000
N	-0.493363000	1.960863000	0.043077000
C	-1.758814000	2.538175000	0.056665000
C	-4.085479000	-1.567935000	-0.125341000
C	-4.372148000	-0.251148000	-0.115818000
C	-3.081658000	0.436526000	-0.110498000
N	-2.060140000	-0.400382000	-0.139099000
C	-2.628699000	-1.673597000	-0.134518000
C	-2.936473000	1.864201000	-0.028644000
N	1.396219000	-3.744915000	0.430671000
N	0.113027000	-4.022261000	0.450054000
C	-0.495890000	-2.923104000	-0.029703000
C	0.451674000	-1.945565000	-0.377956000
C	1.679883000	-2.486749000	-0.042538000
C	-1.930342000	-2.839841000	-0.061536000
C	4.036786000	1.533500000	-0.214070000
C	4.371755000	0.222240000	-0.261813000
C	3.170036000	-0.563723000	-0.083646000
N	2.133256000	0.360795000	0.070252000
C	2.608229000	1.631590000	0.008928000
C	1.855889000	2.794792000	0.103398000
C	2.994322000	-1.910055000	-0.089596000
H	-2.352956000	4.688649000	0.224145000
H	0.287606000	5.140283000	0.316999000
H	-0.426282000	0.957189000	-0.098354000
H	-4.767665000	-2.405241000	-0.121844000
H	-5.339905000	0.227612000	-0.097222000
H	-3.842623000	2.458516000	-0.010321000
H	2.049225000	-4.426901000	0.794017000

H	0.249790000	-1.031273000	-0.902486000
H	-2.478303000	-3.771762000	0.029506000
H	4.686561000	2.386622000	-0.335775000
H	5.348791000	-0.200995000	-0.441387000
H	1.262616000	0.096839000	0.510461000
H	2.409608000	3.724240000	0.113515000
H	3.869984000	-2.547436000	-0.129470000

[PzP-21,22,24-H]+

C	0.672467000	4.142576000	-0.000339000
C	-0.674279000	4.142388000	-0.000311000
C	-1.081061000	2.742702000	-0.000051000
N	-0.000452000	1.911203000	0.000130000
C	1.079663000	2.742958000	-0.000126000
C	4.321757000	-0.705974000	0.000414000
C	4.330034000	0.660700000	0.000368000
C	2.966494000	1.101074000	0.000044000
N	2.187757000	-0.018170000	-0.000113000
C	2.956414000	-1.147789000	0.000131000
C	2.435600000	2.383437000	-0.000150000
N	-0.650474000	-4.052890000	-0.000387000
N	0.652345000	-4.052804000	-0.000337000
C	1.138404000	-2.780415000	-0.000049000
C	0.000595000	-1.823411000	0.000189000
C	-1.136971000	-2.780569000	-0.000121000
C	2.479141000	-2.467792000	-0.000003000
C	-4.330290000	0.658760000	0.000343000
C	-4.321505000	-0.707866000	0.000327000
C	-2.956052000	-1.149054000	0.000054000
N	-2.187984000	-0.019064000	-0.000074000
C	-2.967022000	1.099798000	0.000131000
C	-2.436682000	2.382536000	0.000096000
C	-2.477911000	-2.468777000	-0.000132000
H	1.347836000	4.985217000	-0.000518000
H	-1.349898000	4.984816000	-0.000448000
H	5.171691000	-1.371207000	0.000607000
H	5.188021000	1.315332000	0.000523000
H	1.183901000	0.086393000	-0.000386000
H	3.149732000	3.199034000	-0.000309000
H	0.000623000	-1.199015000	-0.901964000
H	0.000555000	-1.199563000	0.902717000
H	3.201046000	-3.276054000	-0.000185000
H	-5.188668000	1.312886000	0.000509000
H	-5.171329000	-1.373237000	0.000460000
H	-1.184162000	0.085775000	-0.000315000
H	-3.151344000	3.197678000	0.000248000
H	-3.199120000	-3.277661000	-0.000364000

[PzP-21,22,23-H]+

C	-0.232780000	4.164812000	0.123050000
C	1.130027000	4.046472000	0.160371000
C	1.445840000	2.661763000	0.069813000
N	0.268815000	1.972766000	0.005941000
C	-0.795008000	2.856598000	0.022239000
C	-4.347801000	-0.290268000	-0.256639000
C	-4.185173000	1.072696000	-0.320998000
C	-2.819122000	1.367254000	-0.043339000
N	-2.198192000	0.160793000	0.181522000
C	-3.089303000	-0.871361000	0.065838000
C	-2.163038000	2.591382000	-0.048537000
N	0.221450000	-4.097531000	0.028819000
N	-1.055299000	-3.975045000	0.135459000
C	-1.422408000	-2.644047000	0.177389000
C	-0.195911000	-1.804019000	0.112827000
C	0.835214000	-2.861762000	-0.004723000
C	-2.727783000	-2.235962000	0.154864000
C	4.387144000	0.184923000	-0.100231000
C	4.227214000	-1.150539000	-0.148872000

C	2.778536000	-1.390332000	-0.117795000
N	2.092659000	-0.242947000	-0.067294000
C	3.035788000	0.749520000	-0.048392000
C	2.735121000	2.092501000	0.025738000
C	2.184519000	-2.683056000	-0.108414000
H	-0.818206000	5.070441000	0.173851000
H	1.860766000	4.836358000	0.240871000
H	0.290725000	0.977063000	-0.193728000
H	-5.248056000	-0.853516000	-0.448496000
H	-4.930413000	1.809695000	-0.579381000
H	-1.326019000	0.085262000	0.677654000
H	-2.795136000	3.468294000	-0.133148000
H	0.002848000	-1.213337000	1.014454000
H	-0.186223000	-1.135418000	-0.752195000
H	-3.511175000	-2.984201000	0.127181000
H	5.304154000	0.755237000	-0.097767000
H	4.984579000	-1.919169000	-0.192951000
H	3.558943000	2.796476000	0.056252000
H	2.820712000	-3.558894000	-0.168865000

[PzP-2,21,22-H]+

C	1.786571000	3.886872000	-0.000050000
C	0.477981000	4.175641000	-0.000035000
C	-0.235922000	2.880817000	-0.000006000
N	0.629435000	1.850357000	-0.000013000
C	1.866573000	2.418610000	-0.000031000
C	3.950195000	-1.797011000	0.000079000
C	4.346559000	-0.499199000	0.000070000
C	3.158853000	0.317156000	0.000003000
N	2.088643000	-0.511983000	-0.000040000
C	2.504551000	-1.830559000	0.000012000
C	3.062123000	1.711919000	-0.000024000
N	-1.697059000	-3.656032000	-0.000059000
C	0.307244000	-2.933008000	-0.000006000
C	-0.560785000	-1.716454000	0.000041000
C	-1.921234000	-2.324772000	-0.000014000
C	1.716935000	-2.955878000	-0.000011000
C	-3.965033000	1.825946000	0.000051000
C	-4.405468000	0.558488000	0.000043000
C	-3.190123000	-0.282924000	0.000021000
N	-2.075730000	0.465167000	0.000001000
C	-2.484690000	1.745890000	0.000022000
C	-1.641215000	2.849164000	0.000016000
C	-3.145651000	-1.684310000	-0.000015000
H	2.632244000	4.558299000	-0.000067000
H	-0.000227000	5.144169000	-0.000039000
H	4.570939000	-2.680323000	0.000124000
H	5.353719000	-0.111775000	0.000109000
H	1.152656000	-0.102231000	-0.000103000
H	3.990101000	2.270278000	-0.000026000
H	-0.427942000	-1.065484000	-0.871656000
H	-0.427978000	-1.065570000	0.871807000
H	2.206964000	-3.922338000	-0.000045000
H	-4.542426000	2.738876000	0.000067000
H	-5.422906000	0.196552000	0.000050000
H	-2.124694000	3.820485000	0.000032000
H	-4.065121000	-2.257300000	-0.000055000
N	-0.410801000	-4.035713000	-0.000044000
H	-2.401517000	-4.384530000	-0.000096000

[PzP-2,21,23-H]+

C	-0.636962000	4.162679000	0.000462000
C	0.716271000	4.150057000	0.000435000
C	1.146598000	2.770284000	0.000020000
N	0.030163000	1.996725000	-0.000325000
C	-1.097922000	2.791524000	0.000001000
C	-4.368642000	-0.638648000	-0.000362000
C	-4.327603000	0.705697000	-0.000250000

C	-2.901445000	1.063763000	-0.000049000
N	-2.117854000	-0.003859000	0.000311000
C	-2.972306000	-1.088059000	-0.000018000
C	-2.411316000	2.396138000	-0.000157000
N	0.573221000	-3.965463000	0.000221000
N	-0.776797000	-3.993719000	0.000239000
C	-1.175311000	-2.744977000	0.000105000
C	-0.018493000	-1.801438000	-0.000067000
C	1.130403000	-2.742110000	0.000070000
C	-2.549690000	-2.391849000	0.000130000
C	4.341695000	0.629959000	-0.000236000
C	4.341655000	-0.710444000	-0.000217000
C	2.920963000	-1.117171000	-0.000082000
N	2.105815000	-0.040937000	0.000031000
C	2.920645000	1.034210000	-0.000111000
C	2.478714000	2.349842000	-0.000083000
C	2.483060000	-2.442770000	0.000038000
H	-1.295104000	5.018448000	0.000745000
H	1.391640000	4.991796000	0.000707000
H	0.028674000	0.981065000	-0.000761000
H	-5.232024000	-1.287276000	-0.000601000
H	-5.151122000	1.404458000	-0.000436000
H	-3.144964000	3.194377000	-0.000418000
H	1.061162000	-4.853355000	0.000328000
H	-0.006727000	-1.138439000	0.868870000
H	-0.006755000	-1.138703000	-0.869205000
H	-3.275196000	-3.197346000	0.000267000
H	5.183728000	1.306261000	-0.000327000
H	5.183786000	-1.386647000	-0.000282000
H	3.223142000	3.136673000	-0.000131000
H	3.216043000	-3.240662000	0.000131000

[PzP-2,21,24-H]+

C	-0.375508000	4.196243000	-0.000042000
C	-1.691195000	3.925450000	-0.000045000
C	-1.790327000	2.462476000	-0.000020000
N	-0.603225000	1.868489000	0.000016000
C	0.318839000	2.900380000	-0.000001000
C	4.436237000	0.452668000	0.000034000
C	4.009134000	1.727464000	0.000044000
C	2.531646000	1.664944000	0.000017000
N	2.093998000	0.428839000	-0.000013000
C	3.222375000	-0.373998000	0.000020000
C	1.686534000	2.821100000	0.000020000
N	0.425894000	-3.976219000	-0.000040000
N	1.755405000	-3.693328000	-0.000047000
C	1.863039000	-2.394585000	-0.000013000
C	0.517673000	-1.732669000	0.000021000
C	-0.390601000	-2.911047000	-0.000009000
C	3.131185000	-1.734863000	-0.000009000
C	-4.366810000	-0.404939000	0.000061000
C	-3.989447000	-1.703459000	0.000064000
C	-2.537472000	-1.755000000	0.000012000
N	-2.098249000	-0.471531000	-0.000030000
C	-3.166472000	0.404776000	0.000002000
C	-3.037954000	1.762069000	-0.000026000
C	-1.773227000	-2.922560000	-0.000005000
H	-1.154459000	-0.082259000	-0.000083000
H	0.114857000	5.158639000	-0.000060000
H	-2.525057000	4.611519000	-0.000069000
H	5.449780000	0.080462000	0.000042000
H	4.600144000	2.631788000	0.000063000
H	2.206461000	3.774434000	0.000043000
H	0.153608000	-4.951355000	-0.000064000
H	0.388758000	-1.084994000	-0.873742000
H	0.388779000	-1.085053000	0.873828000
H	4.019388000	-2.355901000	-0.000039000
H	-5.369813000	-0.005667000	0.000094000

H	-4.620267000	-2.579189000	0.000099000
H	-3.947221000	2.351312000	-0.000046000
H	-2.307610000	-3.864176000	-0.000022000

[PzP-2,22,23,24-H]2+

C	-0.700191000	4.109699000	-0.495087000
C	0.650709000	4.117442000	-0.495895000
C	1.119799000	2.794326000	-0.147462000
N	-0.009737000	1.986521000	0.012716000
C	-1.156165000	2.780268000	-0.148170000
C	-4.301730000	-0.719297000	0.435336000
C	-4.276444000	0.645496000	0.455429000
C	-2.938275000	1.062857000	0.161393000
N	-2.176980000	-0.046095000	0.027314000
C	-2.972956000	-1.191760000	0.155468000
C	-2.460266000	2.387762000	-0.019602000
N	0.609742000	-3.887834000	-0.617068000
N	-0.691933000	-3.931522000	-0.629410000
C	-1.100623000	-2.763684000	-0.089839000
C	0.013500000	-1.983629000	0.280786000
C	1.124165000	-2.726365000	-0.087857000
C	-2.499108000	-2.464019000	0.030034000
C	4.267855000	0.694893000	0.457988000
C	4.306416000	-0.664938000	0.438497000
C	2.978533000	-1.152857000	0.152227000
N	2.172891000	-0.016196000	0.021423000
C	2.920901000	1.104353000	0.161598000
C	2.434437000	2.417622000	-0.008514000
C	2.521758000	-2.430077000	0.034922000
H	-1.364351000	4.926784000	-0.737885000
H	1.305780000	4.941935000	-0.737599000
H	-0.003680000	1.277694000	0.739044000
H	-5.147889000	-1.362995000	0.629591000
H	-5.097791000	1.317283000	0.654828000

H	-1.308824000	-0.052419000	-0.491925000
H	-3.211826000	3.165645000	-0.080368000
H	1.126378000	-4.667749000	-1.013554000
H	0.017254000	-1.110549000	0.909718000
H	-3.203384000	-3.289345000	0.010149000
H	5.081562000	1.375277000	0.660183000
H	5.157876000	-1.300358000	0.636519000
H	1.306447000	-0.032072000	-0.500689000
H	3.175127000	3.206573000	-0.053140000
H	3.245549000	-3.238363000	0.042803000

[PzP-2,21,22,24-H]2+

C	0.793446000	4.167018000	0.000141000
C	-0.545817000	4.195971000	0.000147000
C	-0.984130000	2.792575000	0.000047000
N	0.049703000	1.936675000	-0.000059000
C	1.173198000	2.747937000	0.000020000
C	4.318969000	-0.787540000	-0.000149000
C	4.350729000	0.571700000	-0.000157000
C	2.987286000	1.033083000	-0.000054000
N	2.182992000	-0.043970000	0.000047000
C	2.935053000	-1.212126000	-0.000012000
C	2.496301000	2.356921000	-0.000048000
N	-0.720961000	-3.974665000	0.000118000
N	0.621342000	-4.041964000	0.000142000
C	1.071162000	-2.813042000	0.000053000
C	-0.059122000	-1.830662000	-0.000054000
C	-1.245332000	-2.734108000	0.000020000
C	2.453121000	-2.495555000	0.000061000
C	-4.315754000	0.811721000	-0.000157000
C	-4.348707000	-0.542003000	-0.000172000
C	-2.980899000	-1.031061000	-0.000054000
N	-2.176635000	0.066634000	0.000041000
C	-2.924339000	1.218537000	-0.000022000

C	-2.372989000	2.472236000	0.000057000
C	-2.578377000	-2.369242000	-0.000007000
H	1.490604000	4.992256000	0.000205000
H	-1.205739000	5.051226000	0.000227000
H	5.157993000	-1.468446000	-0.000216000
H	5.218040000	1.214734000	-0.000240000
H	1.182365000	0.095638000	0.000174000
H	3.242775000	3.142883000	-0.000128000
H	-1.233655000	-4.852972000	0.000186000
H	-0.048097000	-1.205907000	-0.903006000
H	-0.048119000	-1.205733000	0.902781000
H	3.160259000	-3.318096000	0.000152000
H	-5.152680000	1.494612000	-0.000223000
H	-5.217251000	-1.183954000	-0.000252000
H	-1.170727000	0.147582000	0.000171000
H	-3.060958000	3.309953000	0.000133000
H	-3.352677000	-3.126918000	0.000048000

[PzP-2,21,23,24-H]2+

C	1.002109000	4.118493000	0.204809000
C	-0.354383000	4.199951000	0.163258000
C	-0.874952000	2.867948000	0.018124000
N	0.182891000	2.013743000	-0.011387000
C	1.366458000	2.730640000	0.080795000
C	4.288974000	-0.996387000	-0.182276000
C	4.372634000	0.344274000	-0.124287000
C	2.988615000	0.827966000	-0.052837000
N	2.101039000	-0.159278000	-0.065762000
C	2.859121000	-1.319047000	-0.131808000
C	2.633431000	2.207244000	0.042522000
N	-0.894426000	-3.923860000	0.149898000
N	0.447127000	-4.055084000	0.037838000
C	0.942482000	-2.845481000	0.003866000
C	-0.134353000	-1.817725000	0.117378000
C	-1.356252000	-2.670164000	0.183986000
C	2.336633000	-2.586738000	-0.105984000
C	-4.217950000	0.973087000	-0.342565000
C	-4.341441000	-0.373563000	-0.253214000
C	-3.043641000	-0.929106000	0.068634000
N	-2.170256000	0.133186000	0.158543000
C	-2.837808000	1.320457000	-0.076527000
C	-2.252510000	2.560778000	-0.086244000
C	-2.691896000	-2.265978000	0.166257000
H	1.711011000	4.926158000	0.316883000
H	-0.968756000	5.085060000	0.235309000
H	0.210577000	1.031037000	-0.261355000
H	5.088178000	-1.720854000	-0.240768000
H	5.256156000	0.965778000	-0.128129000
H	3.446820000	2.923586000	0.096856000
H	-1.445064000	-4.779004000	0.172316000
H	-0.152388000	-1.145804000	-0.745168000
H	0.032944000	-1.220326000	1.020539000
H	2.998635000	-3.444880000	-0.155806000
H	-4.982938000	1.688225000	-0.608653000
H	-5.226800000	-0.967088000	-0.429052000
H	-1.347864000	0.109550000	0.742018000
H	-2.913497000	3.413005000	-0.193223000
H	-3.483264000	-3.005289000	0.130998000

[PzP-2,21,22,23-H]2+

C	0.463824000	4.192449000	0.142056000
C	-0.888015000	4.144309000	0.172772000
C	-1.285568000	2.758246000	0.067075000
N	-0.134366000	2.009893000	0.003786000
C	0.964838000	2.837928000	0.027519000
C	4.336796000	-0.490954000	-0.250110000
C	4.237886000	0.863410000	-0.344725000
C	2.875767000	1.228732000	-0.065025000

N	2.182705000	0.078623000	0.168918000
C	3.035303000	-1.020712000	0.075138000
C	2.309553000	2.507761000	-0.046815000
N	-0.429561000	-3.995798000	0.039342000
N	0.911324000	-3.982016000	0.131934000
C	1.276022000	-2.721665000	0.180186000
C	0.082376000	-1.817836000	0.120446000
C	-1.027832000	-2.797721000	0.006925000
C	2.638848000	-2.331812000	0.149971000
C	-4.359844000	0.461154000	-0.116131000
C	-4.305831000	-0.876696000	-0.161901000
C	-2.872622000	-1.227742000	-0.115400000
N	-2.093506000	-0.106655000	-0.063879000
C	-2.956593000	0.920215000	-0.055083000
C	-2.579512000	2.273782000	0.021019000
C	-2.391231000	-2.531408000	-0.093225000
H	1.099351000	5.063664000	0.204725000
H	-1.580439000	4.968552000	0.261860000
H	-0.192137000	1.025796000	-0.234155000
H	5.209881000	-1.100772000	-0.433631000
H	5.014655000	1.563510000	-0.614724000
H	1.355006000	0.079190000	0.745474000
H	2.998750000	3.341217000	-0.114632000
H	-0.884002000	-4.904951000	-0.015702000
H	0.102982000	-1.149227000	-0.745355000
H	-0.079960000	-1.210492000	1.018133000
H	3.389457000	-3.113018000	0.100451000
H	-5.227881000	1.104288000	-0.121942000
H	-5.119150000	-1.586062000	-0.210524000
H	-3.370565000	3.014160000	0.054234000
H	-3.097097000	-3.352839000	-0.143067000
